# Neurochemical-hemodynamic-electrophysiological coupling in the neonatal brain: a multimodal MRS-fMRI-EEG investigation

**DOI:** 10.3389/fnins.2026.1859287

**Published:** 2026-07-02

**Authors:** Juliette L. Y. Champaud, Alice R. Thomson, Jucha Willers Moore, Ines Tomazinho, Kathleen Colford, Vyacheslav R. Karolis, Parvaneh Adibpour, Nicolaas A. Puts, Lorenzo Fabrizi, Tomoki Arichi

**Affiliations:** 1 Medawar Pain and Somatosensory Labs, Department of Neuroscience, Physiology and Pharmacology, University College London, London, United Kingdom; 2 Research Department of Early Life Imaging, School of Biomedical Engineering and Imaging Sciences, King’s College London, London, United Kingdom; 3 MRC Centre for Neurodevelopmental Disorders, King’s College London, London, United Kingdom; 4 Department of Forensic and Neurodevelopmental Sciences, King’s College London, London, United Kingdom; 5 Guy’s and St Thomas’ NHS Foundation Trust, London, United Kingdom

**Keywords:** ALFF, brain development, E/I balance, FOOOF, multimodal neuroimaging, neonate

## Abstract

**Introduction:**

Inhibitory and excitatory neurotransmitter levels are linked to fast neuronal oscillations and infra-slow hemodynamic fluctuations, suggesting a shared excitation–inhibition (E/I) regulatory framework across measures. However, these relationships may differ in early development, when both excitatory and inhibitory cortical systems are undergoing substantial functional and structural maturation. Consequently, we hypothesize different functional coupling between neurochemical, electrophysiological, and hemodynamic proxies of E/I signaling in healthy full-term neonates compared to what has been observed in adults.

**Methods:**

Twenty-five healthy full-term neonates (mean postmenstrual age at study = 40.1 ± 1.4 weeks) underwent multimodal MRI and electroencephalography (EEG) recordings during natural resting-state to provide proxy measures of neural excitation and inhibition. These included frontal and occipital MRS measures of *γ*-aminobutyric acid (GABA+) and Glx (glutamate + glutamine) levels, and their ratio; EEG source-reconstructed power spectra decomposed into periodic beta (13–30 Hz) and gamma (30–45 Hz) features (center frequency and peak amplitude), relative to total band power and an aperiodic exponent; and infra-slow fMRI BOLD fluctuations (0.01–0.08 Hz) using amplitude of low-frequency fluctuations (mean and fractional ALFF). Crossmodal relationships were assessed using partial correlations controlling for age.

**Results:**

Occipital GABA+ was negatively correlated with beta relative power (*r* = −0.64, *p* = 0.01) and fractional ALFF (*r* = −0.55, *p* = 0.048), while mean ALFF was negatively correlated with gamma center frequency (*r* = −0.99, *p* = 0.02). These relationships were not observed in the frontal cortex. Instead, frontal Glx positively correlated with beta peak amplitude (*r* = 0.87, *p* < 0.01) and negatively correlated with beta (*r* = −0.78, *p* = 0.02) and gamma (*r* = −0.79, *p* = 0.02) relative power, potentially reflecting the existence of regionally distinct maturational trajectories.

**Discussion:**

Together, these preliminary findings suggest that commonly used neurochemical, oscillatory, and hemodynamic proxy measures of cortical excitatory and inhibitory processes may show only modest correspondence at birth, consistent with ongoing and hierarchal cortical development, leading to complex and asynchronous relationships between these measures.

## 1 Introduction

In the mature adult brain, neuronal processes are commonly assessed using neuroimaging proxy measures of neurochemistry, high-frequency neuronal oscillations, and hemodynamic activity. These measures are widely regarded to be shaped by neuronal excitatory and inhibitory activity, primarily driven by the neurotransmitters glutamate and *γ*-aminobutyric acid (GABA).

High-frequency neuronal oscillations, as measured with electroencephalography (EEG), particularly in the beta (13–30 Hz) and gamma (>30 Hz) bands, have been associated with inhibitory GABAergic neurotransmission and are therefore often considered putative electrophysiological markers of inhibitory processes (Ahmad et al., 2022; Atallah and Scanziani, 2009; Brunel and Wang, 2003). Converging evidence across species supports this link: in rodents, the spiking activity of somatostatin- and parvalbumin-expressing interneurons correlates with cortical beta- and gamma-band power (Chen et al., 2017; Fuchs et al., 2007). In humans, GABA levels, non-invasively measured with Magnetic Resonance Spectroscopy ([^1^H] MRS), have been shown to positively correlate with beta- and gamma-band oscillatory center frequency, peak amplitude and relative power, although effect sizes are variable across regions and paradigms (Balz et al., 2016b; Baumgarten et al., 2016; Cousijn et al., 2014; Edden et al., 2009; Gaetz et al., 2011; Kujala et al., 2015; Muthukumaraswamy et al., 2009) and inversely correlate with the amplitude of spontaneous Blood Oxygen Level-Dependent (BOLD) signal and low-frequency fluctuations (ALFF) (Kurcyus et al., 2018; Muthukumaraswamy et al., 2009). In contrast, MRS-measured glutamate levels inversely relate to gamma peak amplitude (Lally et al., 2014; Whittington et al., 1995). Furthermore, genetic variation in GABA_A_ receptor subunits and pharmacological manipulation of GABAergic signaling has been shown to modulate beta and gamma oscillations (Hall et al., 2010; Jensen et al., 2005; Muthukumaraswamy et al., 2013; Porjesz et al., 2002; Saxena et al., 2013). This suggests that both neurotransmitter availability and signaling may influence neuronal and hemodynamic oscillatory activity, which are themselves correlated (Chang et al., 2013; Leicht et al., 2023; Scheeringa et al., 2011; Tagliazucchi et al., 2012).

Beyond narrowband oscillations, the aperiodic component of the EEG power spectrum (1/f) has emerged as a complementary index of excitation–inhibition (E/I) balance (Donoghue et al., 2020; Gao et al., 2017). The spectral exponent reflects the relative contribution of broadband neuronal activity, with steeper slopes typically interpreted as reflecting greater inhibitory tone and flatter slopes associated with increased cortical excitability (Gao et al., 2017; Lendner et al., 2020). Alterations in this metric have been reported across neurodevelopmental and psychiatric conditions characterized by putative excitation-inhibition imbalance [autism, schizophrenia; (Ellis et al., 2023; Robertson et al., 2019; Salvatore et al., 2024)].

Together, this suggests a framework in which excitation–inhibition balance shapes neuronal activity across multiple scales, with neurochemical, electrophysiological, and hemodynamic measures reflecting shared underlying E/I processes (Fontanier et al., 2022; König et al., 1996; Womelsdorf et al., 2007). Importantly, the neurobiological processes underpinning these measures undergo dramatic, region-specific development along a posterior–anterior gradient over the neonatal period (Gogtay et al., 2004; Kostović et al., 2019) which has clear implications for the establishment of this observed neurochemical-electrophysiological-hemodynamic coupling. Understanding when such associations emerge in development may provide key insights into the spatiotemporal maturation of cortical function across the postnatal period, as disruption during this critical period alters cortical dynamics and confers increased risk of adverse neurodevelopmental outcomes (Eyre et al., 2021; Larroque et al., 2008; Nordvik et al., 2022). Moreover, this knowledge is vital to understand what these commonly used metrics of brain activity represent in early neurodevelopment, and whether they capture the same processes as in the mature brain. To test the hypothesis that significant associations between neurochemical, electrophysiological, and hemodynamic measures are not yet established in the immature neonatal brain, we assessed whether GABA and glutamate levels, fast neuronal oscillations and the aperiodic component, and hemodynamic low-frequency fluctuations (ALFF), within the same cortical regions are correlated in a group of healthy newborns.

## 2 Materials and methods

### 2.1 Subject details

Twenty-five full-term neonates (37.29–43.86 weeks postmenstrual age (PMA), 1–27 postnatal days at scan, 8 female) with no diagnosed brain abnormalities were recruited from the postnatal ward at St Thomas’ Hospital London. Neonates were excluded if there was a history of birth asphyxia, if they were known to have a genetic or metabolic disorder, preterm birth, or any contraindication to MRI scanning such as an implanted medical device. Of the 25, 1 participant was not successfully scanned due to excessive movement and 1 was retrospectively excluded after the unexpected identification of a hypothalamic hamartoma. The final cohort for analysis thus consisted of 23 infants (36.90–41.89 weeks gestational age (GA), 37.29–43.29 weeks postmenstrual age (PMA), 7 female). Studies were performed with UK National Health Service Research Ethics Committee approval (NHS REC: 19/LO/1384) and following written parental consent.

### 2.2 Data acquisition

Electroencephalography, MRS and functional MRI (fMRI) data were collected during natural resting-state. EEG data were acquired for 10–20 min using a 25- or 32- channel Brain Products MR cap system (depending on head size) with a sampling frequency of 5 kHz within 1 h (either before or after) of the MRI scan.

For MRI acquisition, neonates were fed, swaddled, immobilized using a vacuum evacuated blanket (Pearltec, Zurich CH). To provide hearing protection, molded dental putty (President Putty, Coltene Whaledent, Mahwah, NJ, United States) was fitted in the external auditory meatus of the infant’s ear and inflatable cushions were placed over the ears which also minimized head motion (Pearltec, Zurich CH).

BOLD fMRI and MRS data were acquired with a 3T Philips Achieva system with a dedicated neonatal imaging system including a neonatal 32 channel head coil (Hughes et al., 2017) during natural sleep. A high-resolution T_1_-weighted Magnetization-prepared Rapid Gradient Echo (MP-RAGE; resolution: 0.8 mm x 0.8 mm x 0.8 mm, TR/TE: 11/4.6 ms, flip angle: 9°) anatomical image was first acquired for MRS voxel placement and later tissue segmentation (Maria et al., 2021). A high-resolution T_2_-weighted Turbo Spin Echo (TSE; resolution: 0.8 × 0.8 × 1.6 mm; slice gap: −0.8 mm; 125 axial slices; TR/TE: 1200/156 ms, flip angle: 90°) anatomical image was also acquired for later tissue segmentation and image registration.

**Figure 1:**
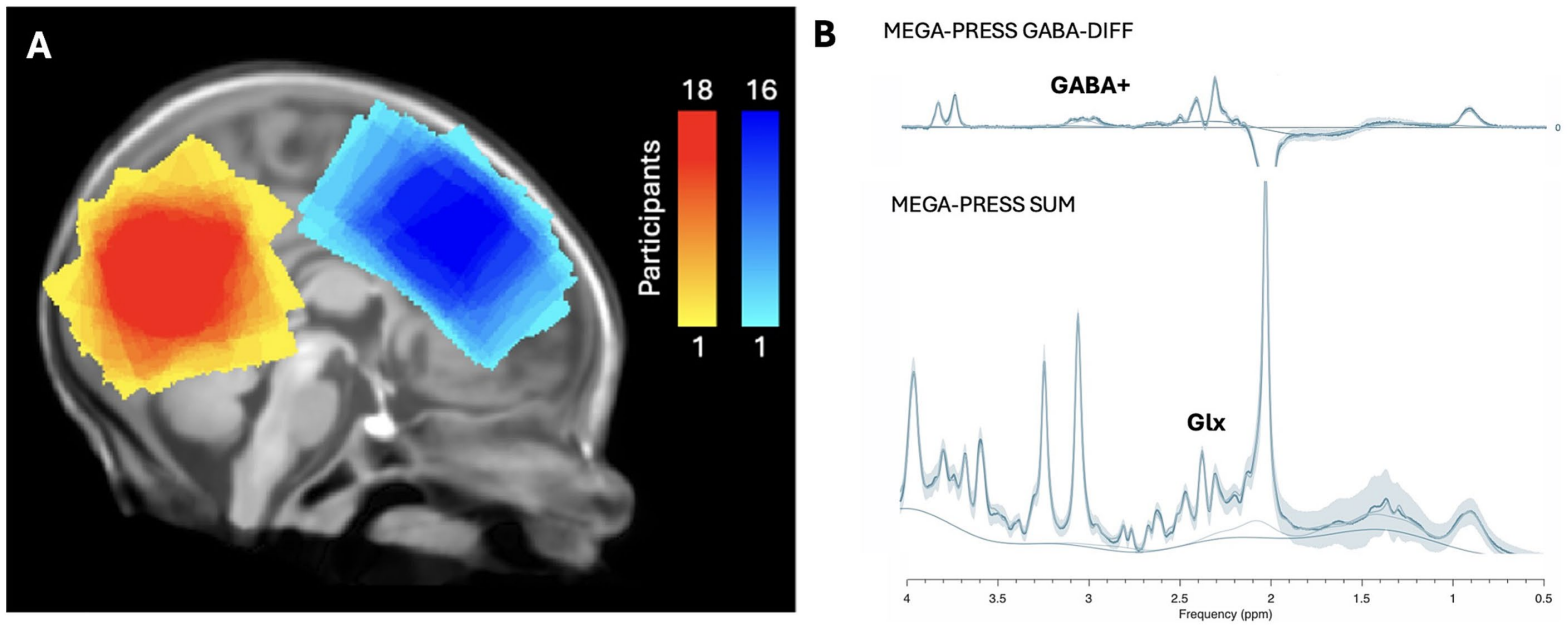
**(A)** Heat maps of MRS voxels in the occipital and frontal regions (27 mL) overlayed on a participant average T_1_ -weighted image. Color scale represents the number of participants per voxel. **(B)** MRS spectrum. Group mean MRS MEGA-PRESS GABA-DIFF and SUM spectra, showing model fit (dark blue line), baseline (light blue lines), and median residuals (blue error bars).

Vendor native MEGA-PRESS (Mescher et al., 1998) [TE 68 ms/TR 2000 ms/240 averages/2000 Hz bandwidth/2048 data points/ using a 1.90 ppm editing pulse (edit-ON) and 7.50 ppm non-editing pulse with CHESS water suppression] were acquired from 27 mL occipital (30 × 30 × 30 mm) and frontal voxels (36 × 30 × 25 mm) voxels (Figure 1). Unsuppressed water acquisitions of MEGA-PRESS were acquired (8 averages) for eddy-current and frequency correction (Marsman et al., 2021; Near et al., 2015).

In 17 of the participants, whole-brain resting-state T2*-weighted single-shot gradient echo echo-planar imaging (GRE-EPI) fMRI data (2 mm isotropic resolution, TR/TE 2930/43 ms) were acquired over 6 min 51 s.

### 2.3 Data processing

#### 2.3.1 Structural data

Individual participant T_2_-weighted images were processed using the neonatal-specific developing Human Connectome Project (dHCP) structural pipeline (Makropoulos et al., 2018), including tissue-type segmentation and cortical surface generation.

A group-specific multimodal structural template was then created from the 11 highest-quality datasets through iterative non-linear alignment with Advanced Neuroimaging Tools (ANTs). In brief, individual participants T_1_- and T_2_-weighted images, cortical gray matter (cGM), white matter (WM) and brainstem segmentations were non-linearly aligned with a T_2_-weighted 40-week PMA neonatal brain template (Schuh et al., 2018), and then averaged to create group-mean T_1_- and T_2_-weighted images, cortical gray matter (cGM), white matter (WM) and brainstem segmentations. Following this, multivariate non-linear alignment was iteratively performed, aligning each participant’s T_1_- and T_2_-weighted structural images, cGM, WM and cerebral spinal fluid (CSF) segmentations data with the group-mean equivalents using ANTs, with each image or segmentation type as separate channels. Once additional iterations of non-linear alignment provided no further improvement, aligned participants T_1_-weighted and T_2_-weighted structural images, cGM, WM and CSF segmentations were averaged to produce a final group-specific multimodal template. Freesurfer’s (8.1.0) *infant_recon_all* tool was then used with the group-specific multimodal template to generate a group-specific cortical surface template.

#### 2.3.2 Magnetic resonance spectroscopy

Raw MRS data were processed using Osprey [2.4.0; (Oeltzschner et al., 2020)], an automated software for MRS analysis in MATLAB (2022a, MathWorks, Inc.) (Harris et al., 2015). We used the following Osprey processing steps: raw data were eddy current-corrected using the water reference followed by frequency and phase correction using robust spectral registration of individual transients (Mikkelsen et al., 2020; Near et al., 2015; Oeltzschner et al., 2020), weighted averaging, Fourier transformation and Hankel Singular Value Decomposition (HSVD) residual water signal removal (Barkhuijsen et al., 1987).

Edit-OFF and Edit-ON spectra were aligned using residual water peaks before Edit-ON and Edit-OFF spectra averages were subtracted to resolve GABA+ (at 3.02 parts per million (ppm)) from the overlaying metabolites (creatine, phosphocreatine, tNAA), which are removed in the resulting GABA difference (GABA-DIFF) spectrum. All averages were combined to create the MEGA-PRESS SUM spectra. Average metabolite spectra were modeled using a TE-specific simulated basis set and a flexible spline baseline available on Osprey and based on vendor, and MRS scan sequence parameters including pulse duration [generated by fast spatially resolved 2D density-matrix simulation in the MATLAB toolbox FID-A; (Oeltzschner et al., 2020; Simpson et al., 2017)]. Basis sets for macromolecule and lipid contributions were integrated as Gaussian basis functions (Oeltzschner et al., 2020). DIFF and SUM spectra were modeled between 0.5 ppm and 4 ppm with linear baseline correction and a knot spacing of 0.55 ppm according to the Osprey model algorithm (Oeltzschner et al., 2020). Modeling was performed for 19 metabolites (ascorbic acid, aspartic acid, total creatine, creatine methylene, GABA+, glycerophosphocholine, glutathione, glutamine, glutamate, myo-inositol, lactate, total N-acetylaspartate, N-acetylaspartylglutamate, total choline, phosphocholine, phosphocreatine, phosphatidylethanolamine, scyllo-inositol, taurine), 5 macromolecules and 3 lipids (MM09, MM12, MM14, MM17, MM20, Lip09, Lip13, Lip20) for both SUM and DIFF spectra. Glx (at 2.1–2.4 ppm) were quantified from the SUM spectra, while GABA+ was quantified from the MEGA-PRESS GABA-DIFF spectra (Figure 1). A proximal measure of ‘E/I’ ratio, Glx: GABA+, was calculated for each participant in each region of interest (ROI; frontal and occipital).

The Osprey co-registration module (via SPM version 12) was used to register MRS acquisition voxels to the participants’ own T_2_-weighted images. Segmented T_2_ images were used to obtain tissue-corrected water-scaled estimates of metabolite concentrations (IU), whereby concentrations are scaled according to the assumption that metabolite concentrations in CSF are negligible (Gasparovic et al., 2006; Harris et al., 2015). Water-scaled metabolite concentrations were then corrected for differences in metabolite and water T_1_ and T_2_ relaxation times (derived from literature values; see Supplementary Table 1), as well as for differences in water content across GM, WM, and CSF, following established methods (Gasparovic et al., 2006; Oeltzschner et al., 2020). “Alpha correction” of tissue-corrected GABA+ concentrations was then performed, with the assumption that GABA+ concentration is two times more concentrated in GM compared to WM (Harris et al., 2015; Jensen et al., 2005).

Spectra were visually assessed by an experienced MRS data user (AT). MRS spectra with significant artifacts due to motion and/or lipid contamination and/or spurious echoes were excluded. As per MRSinMRS (Lin et al., 2021), quantitative quality metrics (QM) were assessed including signal to noise ratio (SNR) of the creatine peak, linewidth of the creatine peak [full-width-at-half-maximum (FWHM)], residual fit of spectra to basis sets, and the frequency shift used for spectral alignment. The Osprey model algorithm (Osprey Version 2.4.0; Oeltzschner et al., 2020), utilized for modeling the MEGA-PRESS SUM and GABA-DIFF spectra, currently does not provide individual neuro-metabolite QM. Reported QM are thus based on average model fits of the MEGA-PRESS spectra (Supplementary Table 2). The resulting sample of MRS data consisted of 21 participants.

To extract region-specific metrics for cross-modality comparisons, the MRS voxels were used to define anatomical ROIs by registering participants’ own MRS voxels to the group-specific template. For EEG-fMRI analysis in participants where MRS data was not successfully acquired, group-level average MRS voxel masks were used (Supplementary Figure 1).

#### 2.3.3 Functional magnetic resonance imaging

Resting-state BOLD fMRI data were successfully acquired from 17 participants and were pre-processed as follows: skull striping using FSL’s Brain Extraction Tool [BET; (Smith, 2002)], motion correction by spatial alignment of each volume to the reference (middle volume of the time-series) using FreeSurfer’s implementation of AFNI’s 3DVolreg (Cox, 1996). EPI distortion correction was performed using FSL’s TOPUP tool with a reverse phase encoding image (Andersson et al., 2003). Distortion corrected fMRI data were aligned to the group-specific T_2_-weighted template (resampled to match the spatial resolution of the fMRI data) by concatenating affine and non-linear spatial registrations via the participants’ own T_2_-weighted structural data using FreeSurfer (Reuter et al., 2010) and ANTs tools (Avants et al., 2011). Timeseries data were detrended using AFNI’s 3dTproject (Cox, 1996), including 24 motion parameters (Satterthwaite et al., 2013). The group mean absolute displacement (AD) was 0.24 mm (range: 0.05–0.68 mm), and maximum AD was 1.55 mm (range: 0.18–3.54 mm). The group mean framewise displacement (FD) was 0.24 mm (range: 0.04–0.68 mm) and maximum FD was 5.24 mm (range: 0.21–12.93 mm). To further mitigate the effects of head motion on the BOLD signal and further analyses, zero censoring of high-motion volumes (FD > 0.3 mm and AD > 1 mm) was applied, with 12.71% (range: 0–34.67%) mean number of censored volumes.

Infra-slow BOLD fluctuations were quantified in the 0.01–0.08 Hz range (Supplementary Figures 2B,C) using AFNI’s 3dRSFC tool (Cox, 1996). To assess the relationship with neurochemical regulation and fast neuronal oscillations, mean and fractional ALFF (mALFF and fALFF) values were calculated for each participant in the MRS acquisition voxel ROIs (frontal and occipital). To do this, voxelwise ALFF values within each MRS acquisition voxel were averaged and then normalized by the global mean ALFF to minimize intersubject differences in overall signal amplitude (mALFF) (Zang et al., 2007). Fractional ALFF (fALFF) was computed as the ratio of the power within the low-frequency band (0.01–0.08 Hz) to the total power across the full frequency range, providing a normalized index of the relative contribution of slow fluctuations to the overall BOLD signal (Zou et al., 2008).

#### 2.3.4 Electroencephalography

##### 2.3.4.1 Pre-processing

**Figure 2:**
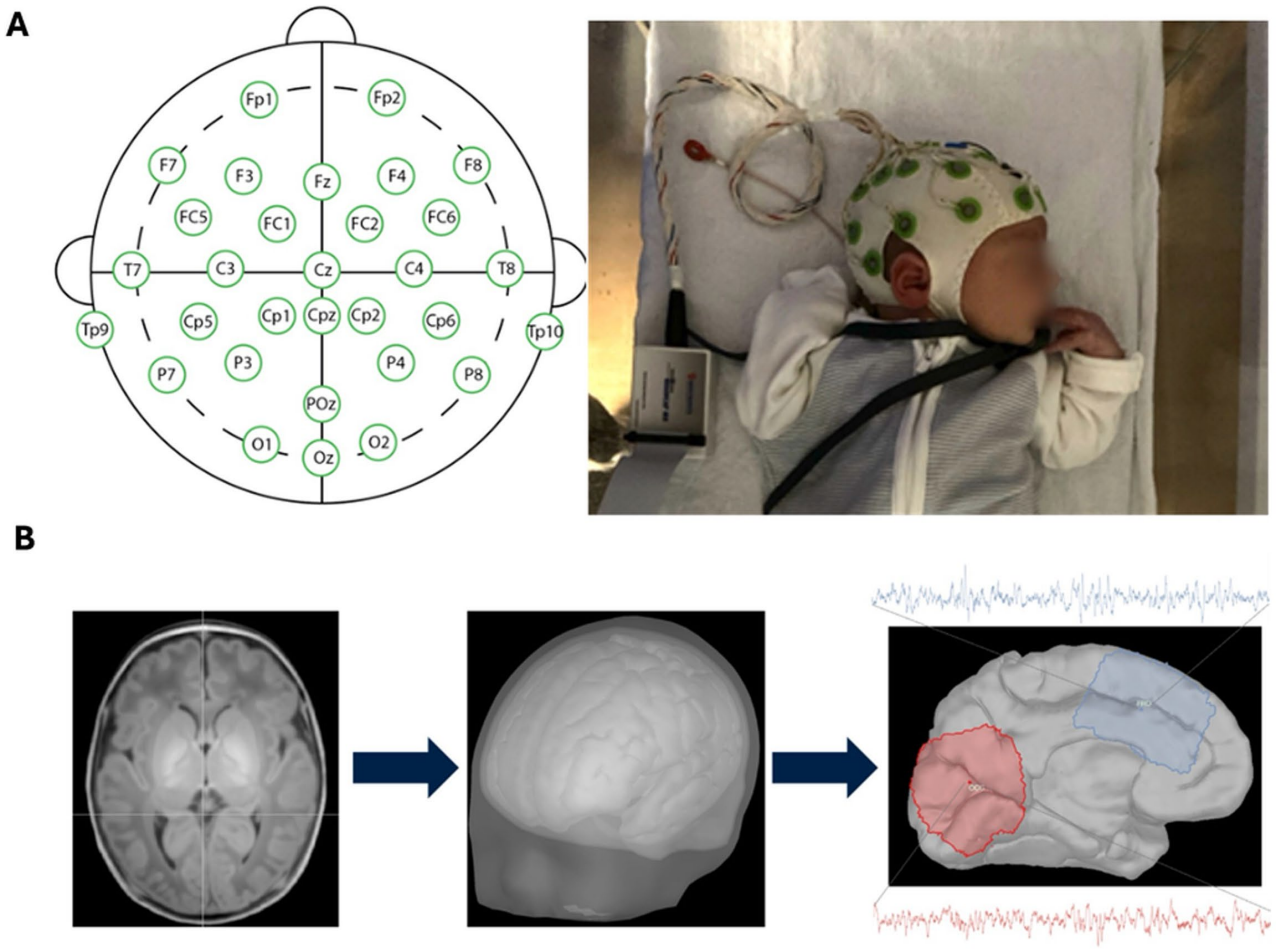
EEG acquisition and virtual electrode analysis **(A)** EEG electrode placement, which included electrodes over the primary visual (Oz, O1, O2), auditory (T7, T8), association (POz, Fz, F7, F3, F4, F8, TP9, TP10, P7, P8, CP5, CP6, FC5, FC6, Fp1, Fp2, P3, P4) and somatosensory cortices (FC1, FC2, C3, Cz, C4, CP1, CP2, CPz). Image shows a neonate at 37 weeks gestational age wearing a 32-channel Brain Products MR Cap. **(B)** The group-specific T_1_-weighted template was used to generate a BEM of scalp, outer/inner skull and cortical surface. MRS voxels were intersected with cortical surface and PCA-reduced virtual timeseries were extracted from these locations, using Brainstorm – average voxel and participant virtual timeseries shown.

EEG data were pre-processed using MATLAB (2021a, MathWorks, Inc.) and EEGLAB (Delorme and Makeig, 2004). Raw data were filtered with a notch (48–52 Hz) and second-order bidirectional Butterworth bandpass filter (0.1–70 Hz). A single epoch of 120–150 s free of gross artifacts (visually assessed by an experienced EEG user, JC) was selected. Channels deemed to have poor quality recording, due to poor contact with the scalp, assessed visually by JC (e.g., non-recording electrodes producing flat signal, elevated high-frequency noise, repeated instances of high amplitude activity referred to as ‘electrode pops’), were rejected (0–2 channels in each epoch). The remaining data were denoised using independent component analysis (ICA) [0–3 discrete ICs were removed corresponding to ECG breakthrough, muscle, eye movement or equipment artifacts in conjunction with the ICLabel prediction tool (Pion-Tonachini et al., 2017)]. Spherical interpolation was then used to estimate data from non-recording channels, poor-quality channels and channels that could not be denoised (0–8 in all datasets). A consistent 31-channel EEG montage was used across recordings, with channels interpolated or excluded as needed to match the configuration (9 channels interpolated for 4 participants with the 25-channel cap; the number of interpolated channels including poor-quality ones never exceeded 10 and were distributed across the scalp) (Figure 2A). Pre-processed data were downsampled to 500 Hz and re-referenced to the common average. EEG data were collected from 22 out of 23 participants, however 4 were excluded due to poor data quality, resulting in a final EEG sample size of 18 participants.

Sleep–wake states were defined using EEG, heart rate, respiratory, and cot side behavioral monitoring. EEGs were manually scored by an experienced EEG user (JC) as wakefulness, active (AS) or quiet sleep (QS) in 30-s epochs, according to the criteria of the American Academy of Sleep Medicine for infants (Grigg-Damberger, 2016). Of the 18 participants, 11 were in AS, 2 in QS, and 5 awake. Because of the small and unevenly distributed sleep-state groups, vigilance state was summarized descriptively but was not included as a statistical covariate.

##### 2.3.4.2 Source reconstruction

Source reconstruction was performed for each participant using Brainstorm (Tadel et al., 2011) using the previously-created common neonatal template head model (see 2.2.1 Structural data) to estimate cortical voltage activity within the ROIs: frontal and occipital MRS voxels. Anatomical surfaces were first generated from the group-template T1-weighted MRI volume (Figure 2B) using a boundary element method (BEM) approach (He et al., 1987). The template was segmented to delineate the scalp, outer skull, inner skull, and cortical surfaces, which form the basis of a realistic three-layer boundary element head model. To approximate neonatal anatomy, the skull thickness parameter was reduced from the Brainstorm default of 4 mm to 2 mm, reflecting the thinner neonatal skull (Li et al., 2015). Electrodes were then registered to the generated scalp surface using standard 10–5 channel locations and the Brainstorm “project electrodes to surface” tool.

Following surface generation and electrode registration, a three-layer BEM forward model was constructed (Figure 2B), using the OpenMEEG software package (Gramfort et al., 2010; Kybic et al., 2005). Because neonatal skull conductivity is higher than adult skull conductivity, tissue conductivities were adjusted based on neonatal EEG modeling literature (McCann et al., 2019; Odabaee et al., 2014): scalp: 0.33 S/m; skull: 0.13 S/m, and combined brain + cerebrospinal fluid (CSF) compartment: 0.33 S/m. Source dipole locations were constrained to the gray matter surface with orientations fixed perpendicular to the cortical surface (Dale et al., 1993).

Minimum Norm Estimation (MNE) (Hämäläinen and Ilmoniemi, 1994) with dynamic Statistical Parametric Mapping (dSPM) (Dale et al., 2000) was used to estimate the activation sources. A non-normalized identity matrix is used as the noise covariance matrix for dSPM, assuming identical noise levels across EEG channels, due to the resting-state nature of the data. EEG sources were estimated for each participant at all time points.

##### 2.3.4.3 Frequency analysis

Power spectral densities (PSDs) were computed from the virtual electrode time-series using Welch’s method with 2-s segments and 50% overlap (Cohen, 2014; Welch, 1967). The resulting average PSDs across participants in each ROI are plotted in Supplementary Figure 4A. The resulting PSDs were then analyzed using the Fitting Oscillations and One-Over-F (FOOOF) algorithm, now called SpecParam model, which parameterizes electrophysiological data into its aperiodic and periodic components (Donoghue et al., 2020). Model parameters were selected based on previously reported parameters, maximizing the model fit across participants (minimum *R*^2^ value of 0.85), regardless of the presence, or not, of periodic oscillations (Luotonen et al., 2025; Rico-Picó et al., 2023): *peak_width_limits:* [1.5, 12]; *max_n_peaks:* 5; *min_peak_height:* 0; *peak_threshold:* 2; *aperiodic_mode:* fixed. The model was fit on 0.5–45 Hz range to avoid the significant challenge of accurately fitting spectral peaks around the 50 Hz line noise artifact and aligning with the trend of reduced high-frequency (>50 Hz) oscillatory power during early brain development (Stanyard et al., 2024).

The following metrics were extracted for every participant and each ROI: broadband aperiodic exponent, periodic components, defined by center frequency and peak amplitude, and relative power within the predefined bands of interest: beta, 13–30 Hz, and gamma, 30–45 Hz. *Center frequency* was defined as the frequency corresponding to the *peak amplitude* within a given frequency band. *Relative power* was calculated as the area under the PSD curve within that frequency band (using trapezoidal numerical integration), normalized by the total area under the full PSD. These components were selected for consistency with previous studies combining EEG and MRS (Balz et al., 2016a; Baumgarten et al., 2016; Edden et al., 2009; Gaetz et al., 2011; Kujala et al., 2015; Muthukumaraswamy et al., 2009). All participants’ periodic components of center frequency and peak amplitude from each ROI are plotted in Supplementary Figure 4B.

These frequency bands were selected *a priori* because previous adult studies have linked beta and gamma activity to MRS-derived neurochemical markers, haemodynamic measures, and excitation–inhibition-related processes (Ahmad et al., 2022; Atallah and Scanziani, 2009; Brunel and Wang, 2003).

### 2.4 Statistical analysis

Statistical analyses were performed in MATLAB (2021a, MathWorks, Inc.). Demographic effects were assessed by calculating linear correlation coefficients (Pearson’s rho) between PMA/postnatal age (PNA)/sex and each measure of interest for both occipital and frontal ROIs. To investigate the relationships between neurotransmitter, electrophysiological and hemodynamic correlates of E/I, linear partial correlation coefficients (adjusting for PMA and PNA), for both occipital and frontal ROIs, between MRS and EEG, MRS and fMRI, and EEG and fMRI measures were computed. The relationships examined were informed by previously reported associations in adult cohorts linking neurochemical, electrophysiological, and hemodynamic measures to aspects of excitation–inhibition processes and were selected to test whether similar cross-modal relationships are observable in early development (Table 1). Due to the exploratory nature of this study, the following results are reported without correction for multiple comparisons. Since not all participants with MRS data also had EEG or fMRI data, the sample size for each comparison differed across modality and ROI, which is indicated explicitly in the result tables presented in the Supplementary material.

**Table 1:** Table illustrating the correlations assessed in this work, selected to capture complementary indices of excitation–inhibition processes across neurochemical (MRS), electrophysiological (EEG), and hemodynamic (fMRI) domains.

Measures	GABA+	Glx	Glx/GABA+	BOLD ALFF metrics	Rationale
EEG periodic and relative power metrics	×	×		×	Assess how local neurochemical composition (GABA+, Glx) and infra-slow activity (ALFF) relate to the amplitude and frequency of fast oscillatory dynamics.
EEG aperiodic exponent			×		Assess how the balance of excitation and inhibition shapes scale-free, broadband electrophysiological activity.
BOLD ALFF metrics	×	×			Examine how local neurochemical concentrations (GABA+, Glx) relate to the amplitude of intrinsic infra-slow hemodynamic fluctuations.

## 3 Results

### 3.1 Final sample sizes

Sample sizes differ across modalities due to infants awakening during scanning preventing data acquisition of all modalities, or head motion resulting in data of a single modality being excluded following strict modality-specific quality control measures. Following data quality checks and model fits, final sample sizes are reported in Supplementary Table 3. The FOOOF model identified a gamma-range peak in 22% of frontal ROIs and 33% occipital ROIs. Beta peaks were identified in 89% of frontal ROIs and 100% of occipital ROIs.

### 3.2 Demographic effects

No significant correlations were observed between PMA and EEG aperiodic exponent, periodic components (center frequency, peak amplitude) and relative power, or GABA+ and Glx levels, and their ratio (Glx/GABA+) (Supplementary Figures 5, 6). A significant positive relationship was observed between PNA and beta center frequency (*r* = 0.47, *p* = 0.048), GABA+ level (*r* = 0.67, *p* = 0.02) and Glx level (*r* = 0.76, *p* = 0.007) (Supplementary Figures 8, 9). A significant negative relationship was observed between age and mALFF (PMA, frontal ROI only: *r* = −0.56, *p* = 0.02; PNA, frontal ROI: *r* = −0.86, *p* < 0.001; PNA, occipital ROI: *r* = −0.74, *p* < 0.001), while no significant relationship was found between age and fALFF (Supplementary Figures 7, 10). No significant differences were observed between male and female in any measure of interest (Supplementary Figures 11–13). All linear correlation values are reported in Supplementary Tables 4–6.

### 3.3 Cross-modal analysis

#### 3.3.1 Occipital GABA+ negatively correlates with beta relative power, while frontal Glx negatively correlates with beta and gamma relative power, and positively with beta peak amplitude

**Figure 3:**
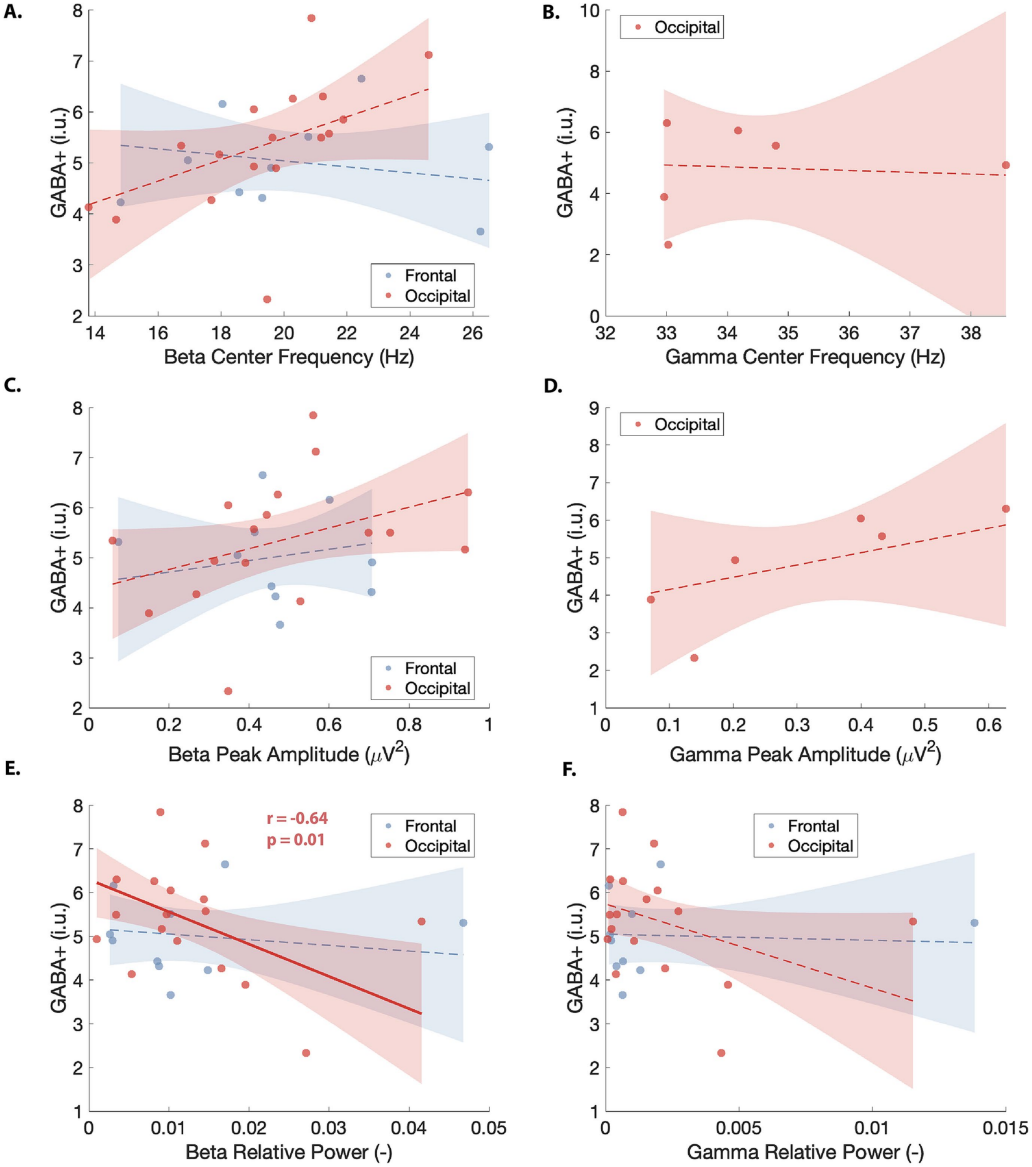
GABA+ level negatively correlated with beta relative power in the occipital ROI. Scatter plots with overlayed regression lines correlating GABA+ to EEG beta and gamma center frequency **(A,B)**, peak amplitude **(C,D)** and relative power **(E,F)**. Solid line: *p* < 0.05. **(A,C,E,F)** Frontal ROI: *n* = 10 participants, occipital ROI: *n* = 10 participants, and **(B,D)** occipital ROI: *n* = 6 participants.

**Figure 4:**
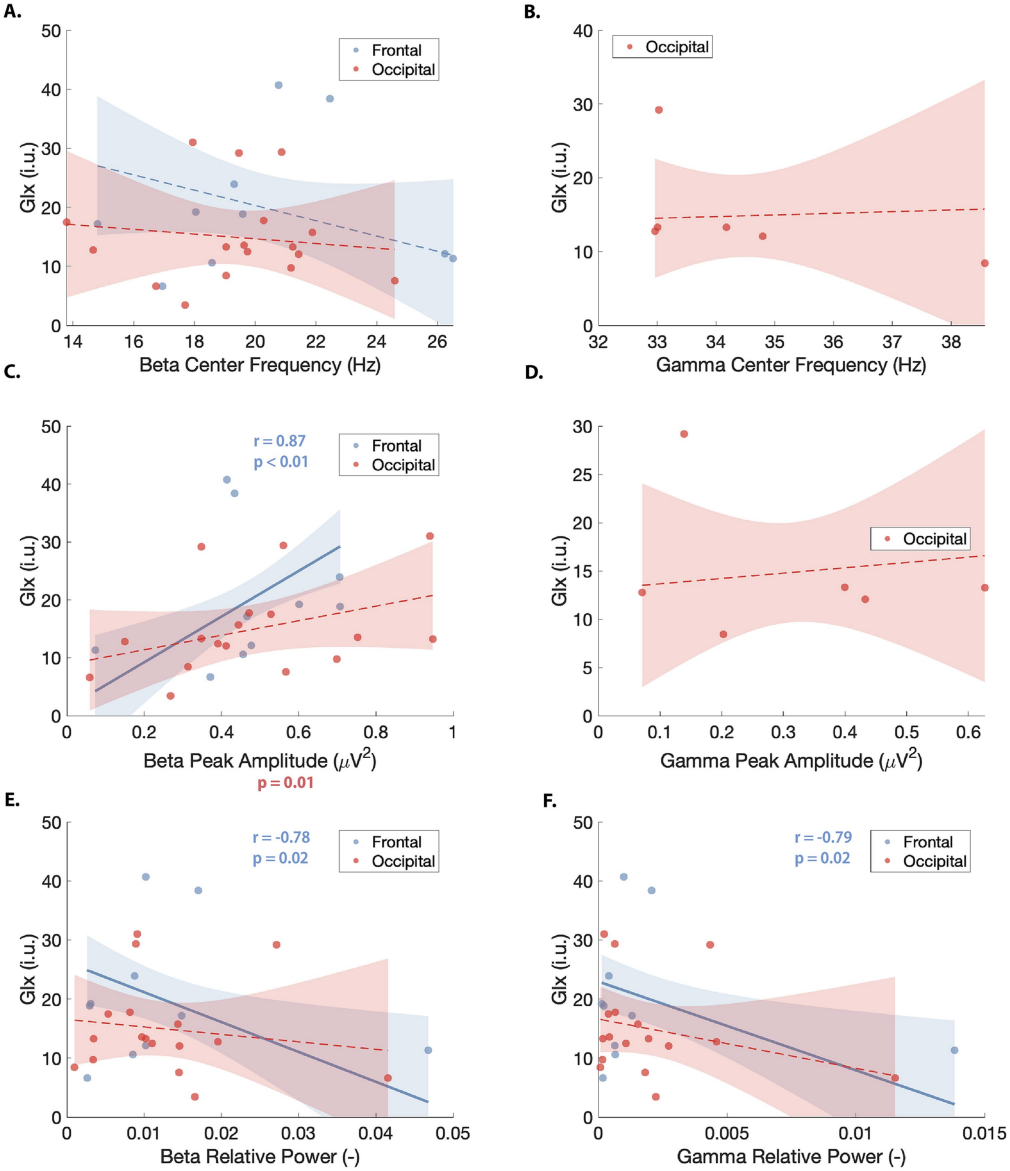
Glx level positively correlated with beta peak amplitude and negatively with beta and gamma relative power in the frontal ROI. Scatter plots with overlayed regression lines correlating Glx to EEG beta and gamma center frequency **(A,B)**, peak amplitude **(C,D)** and relative power **(E,F)**. Solid line: *p* < 0.05. **(A,C,E,F)** Frontal ROI: *n* = 10 participants, occipital ROI: *n* = 17 participants, and **(B,D)** occipital ROI: *n* = 6 participants.

GABA+ level was negatively correlated with beta relative power in the occipital ROI (r = −0.64, *p* = 0.01) (Figure 3) but had no relationship with any EEG metrics in the frontal ROI. On the other hand, Glx level was positively correlated with beta peak amplitude and negatively correlated with beta and gamma relative power in the frontal ROI (*r* = 0.87, *p* < 0.01; *r* = −0.78, *p* = 0.02; *r* = −0.79, *p* = 0.02) (Figure 4) but had no relationship with any EEG metrics in the occipital ROI. All partial correlation values are reported in Supplementary Table 7. Correlations were not tested for gamma center frequency and peak amplitude, due to insufficient peaks in that frequency range in the frontal ROI.

#### 3.3.2 No relationship between Glx/GABA+ ratio and EEG aperiodic exponent

**Figure 5:**
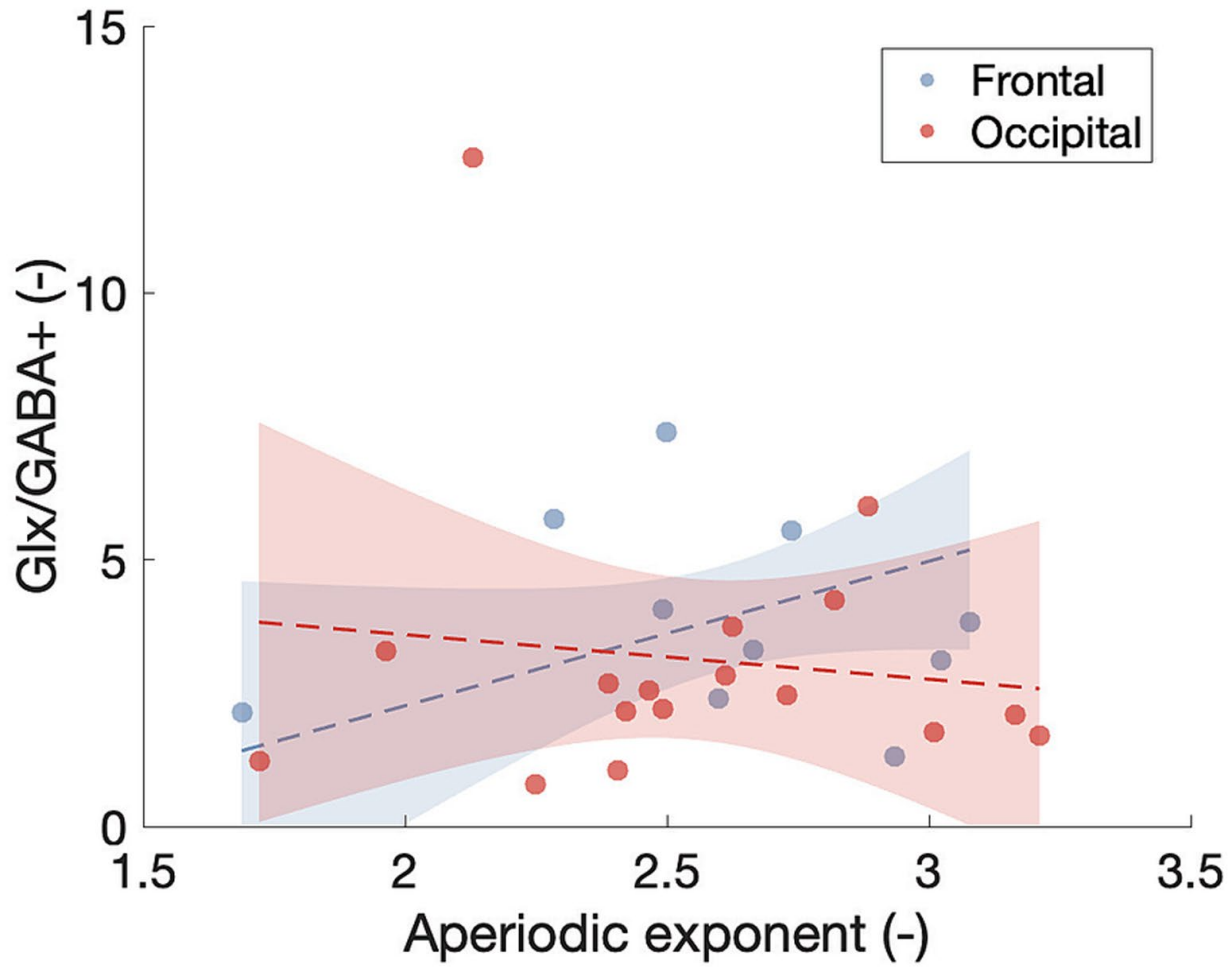
Glx/GABA+ and EEG aperiodic exponent have no relationship in either ROI. Scatter plots with overlayed regression lines correlating Glx/GABA+ to EEG aperiodic exponent. Frontal ROI: n = 10 participants and occipital ROI: *n* = 17 participants.

No relationship was found between Glx/GABA+ ratio and EEG aperiodic exponent in frontal or occipital ROIs (Figure 5 and Supplementary Table 8).

#### 3.3.3 Fractional ALFF negatively correlated with GABA+ level

**Figure 6:**
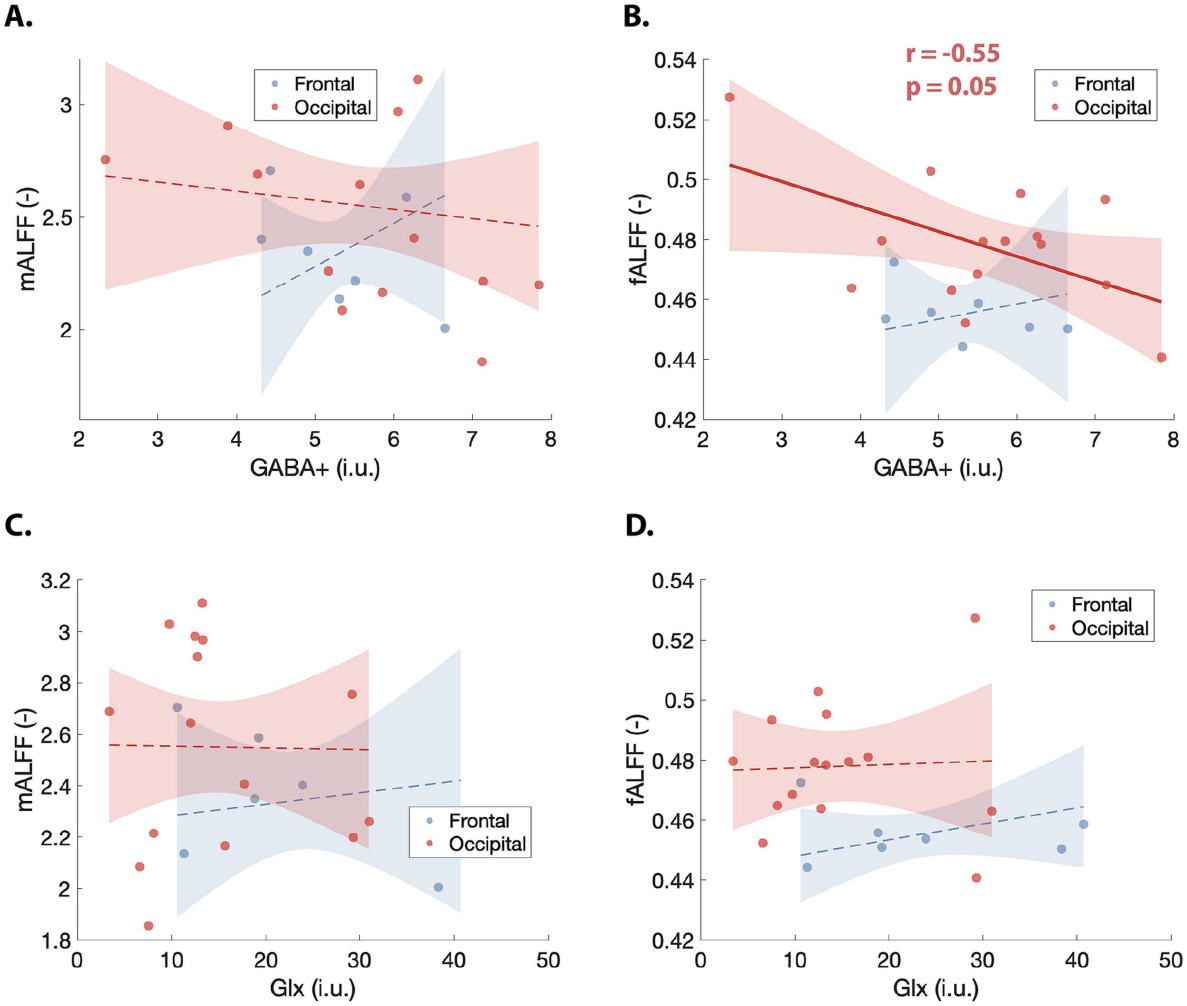
GABA+ level negatively correlated with fALFF. Scatter plots with overlayed regression lines correlating GABA+ **(A,B)** and Glx **(C,D)** to mALFF and fALFF. Solid line: *p* < 0.05. Frontal ROI: *n* = 7 participants and occipital ROI: *n* = 15 participants.

GABA+ level was negatively correlated with fALFF in the occipital ROI (*r* = −0.55, *p* = 0.05) (Figure 6) but had no relationship with mALFF. GABA+ level had no relationship with fALFF or mALFF in the frontal ROI. Glx level had no relationship with fALFF or mALFF in either ROI. All partial correlation values are reported in Supplementary Table 9.

#### 3.3.4 Mean ALFF negatively correlates with gamma center frequency

**Figure 7:**
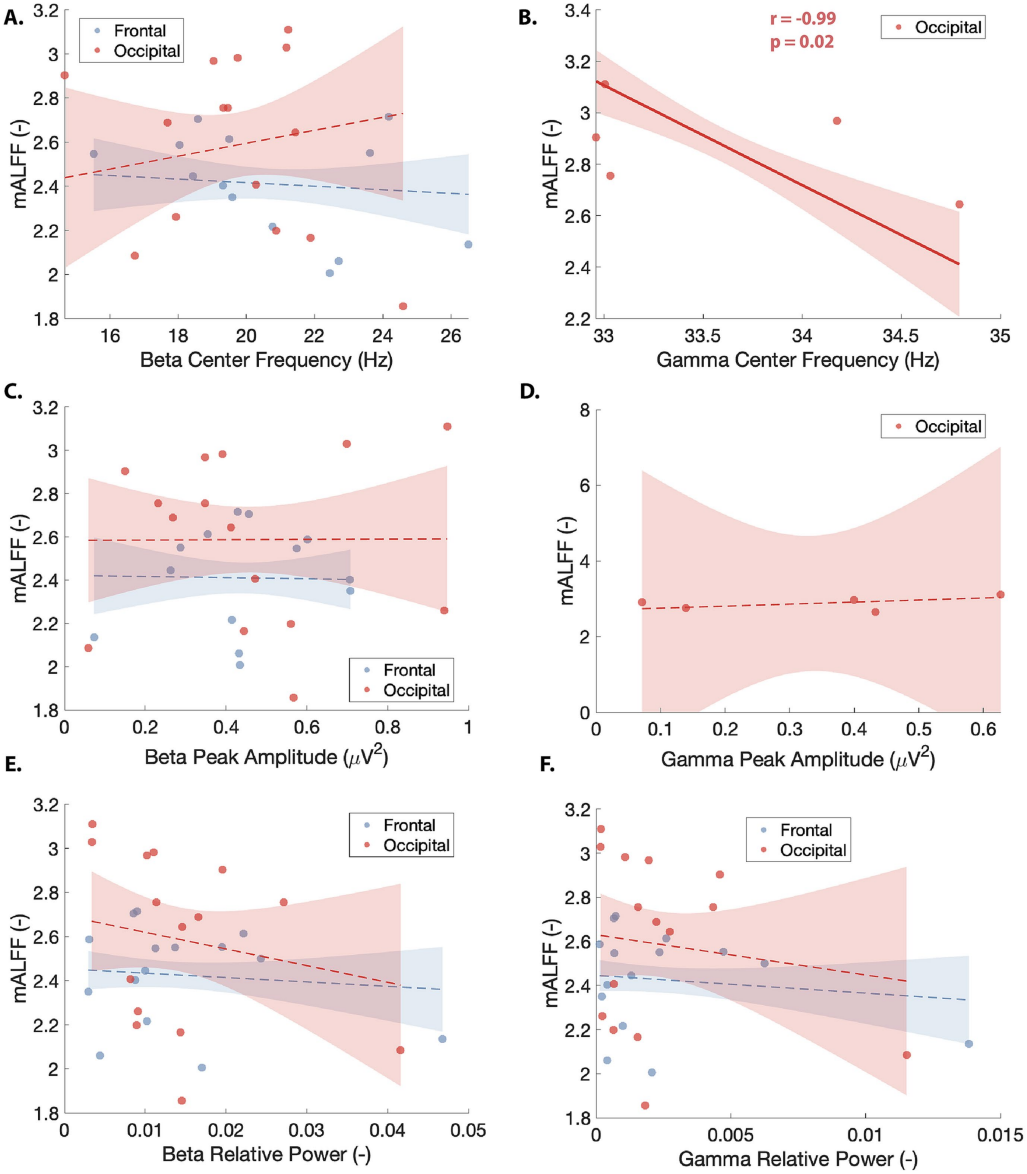
mALFF negatively correlated with gamma center frequency. Scatter plots with overlayed regression lines correlating mALFF to EEG beta and gamma center frequency **(A,B)**, peak amplitude **(C,D)** and relative power (**E,F**; *n* = 15 participants for frontal and occipital ROIs). **(A,C)** Frontal ROI: *n* = 13 participants, occipital ROI: *n* = 15 participants, and **(B,D)** occipital ROI: *n* = 5 participants.

Mean ALFF was negatively correlated with gamma center frequency in the occipital ROI (*r* = −0.99, *p* = 0.02) (Figure 7) but had no relationship with any EEG metrics in the frontal ROI. fALFF had no relationship with any beta or gamma periodic or relative power measures in both frontal and occipital ROIs (Supplementary Figure 8). All partial correlation values are reported in Supplementary Table 10.

## 4 Discussion

Fast neuronal and slow hemodynamic oscillations are related to excitatory and inhibitory processes in adults. However, the neurobiological infrastructure supporting these associations develops dramatically in the postnatal period. Here, we investigated the relationship between MRS-measured inhibitory GABA and excitatory Glx levels in frontal and occipital brain regions, EEG-measured beta and gamma neuronal oscillations as well as aperiodic activity, and fMRI measured ALFF in the same regions and healthy full-term neonates.

**Figure 8:**
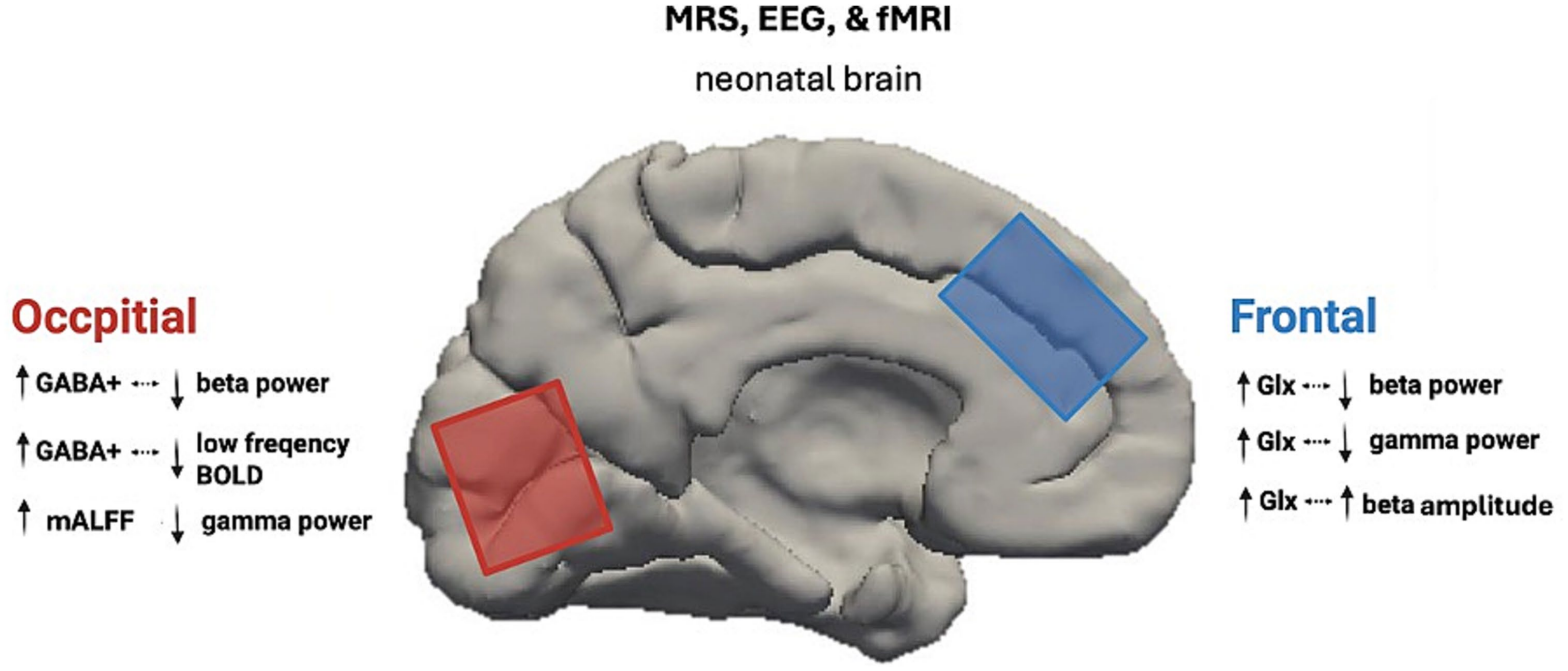
Summary diagram of the regional relationships observed between neurochemical, electrophysiological and hemodynamic measures.

Our preliminary results show only limited associations, primarily linking high-frequency EEG activity, low-frequency BOLD fluctuations, and GABA+ levels within the occipital cortex (Figure 8). This suggests that high-frequency neural activity and slow hemodynamic fluctuations may already reflect some properties of cortical inhibitory neurochemical levels. An important consideration is that the absence of clear associations here does not necessarily imply that these measures are completely independent in the neonatal brain, rather that the cortical structure and function supporting them is undergoing asynchronous and non-linear development, likely leading to more complex relationships not detectable with the approaches used here. In the mature adult brain, cortical organization and functionality is comparably stable, making the relationship between these measures more robust.

The neurobiological processes underpinning each measure examined here are likely undergoing rapid maturation during the neonatal period. Consistent with this, neonatal EEG is characterized by discontinuous, low-frequency activity with limited high-frequency power, and elevated aperiodic exponents relative to later developmental stages (Chini et al., 2022; Khazipov and Luhmann, 2006; Stanyard et al., 2024). In parallel, resting-state network strength and organization, and BOLD signal amplitude, including ALFF, undergo substantial postnatal development (Doria et al., 2010; Fransson et al., 2007; Wu et al., 2016). Limited MRS evidence also suggests an age-related increase in GABA+, with lower basal ganglia and cerebellar GABA+ levels reported in neonates (35–43 PMA weeks) compared to children [6–16 years (Tomiyasu et al., 2017)]. Against this background of ongoing cortical maturation, our findings suggest that the neurobiological drivers of common measures of neurochemistry, electrophysiology, and hemodynamics at term age may differ from those reported in adults, resulting in limited evidence of consistent coupling across modalities in the present sample.

Consistent with our hypothesis, there was no association between the aperiodic exponent and the Glx/GABA ratio in either cortical region. This contrasts with findings in adults, where steeper aperiodic slopes (higher exponents) have been associated with stronger inhibitory tone, yielding a negative relationship with E/I ratio (Gao et al., 2017; Lendner et al., 2020). The absence of a similar relationship in the present neonatal sample may reflect the developmental immaturity of inhibitory circuitry and its control over cortical excitation. This would be in keeping with work suggesting that GABA may exert depolarizing effects during early postnatal life, particularly in later-maturing regions such as frontal cortex, meaning that mature electrophysiological signatures of E/I balance may not yet apply (Ben-Ari, 2002; Peerboom and Wierenga, 2021). In addition, GABAergic interneurons are known to mature during the third trimester, where they undergo prolonged migration and maturation, with continued integration into cortical circuits only after birth [as reviewed by Marín (2025)]. In parallel, thalamocortical glutamatergic synapses onto cortical PV + interneurons have been shown to promote inhibitory circuitry myelination and constrain cortical plasticity (Stedehouder and Kushner, 2016; Sydnor et al., 2025; Wu et al., 2024). These developmental processes occur across the cortex hierarchically: thalamocortical projections to sensory regions are established earlier in the third trimester, maturing postnatally, while thalamocortical projections to the frontal and parietal cortices continue to mature into adolescence and adulthood (Kostović et al., 2019; Sydnor et al., 2025). Thus, the balance of cortical excitatory and inhibitory processes that may typically shape the aperiodic exponent in the mature adult brain may be immature during the period studied. However, given the exploratory nature of the present analyses and modest sample size, this requires replication in larger developmental cohorts.

Consistent with the finding that electrophysiological signatures of excitation–inhibition balance may not yet be established at birth, GABA+ levels failed to associate with high-frequency electrophysiology metrics, which has been observed in adults during auditory tasks and at rest (Balz et al., 2016a; Baumgarten et al., 2016). As discussed above, the nature of GABAergic activity in the early neonatal period is potentially different, and its influence on neural dynamics may reflect immature or regionally heterogeneous circuit properties rather than the stable inhibitory control observed in adults. In contrast, Glx levels positively correlated with beta peak amplitude, suggesting that higher Glutamate tone is associated with a greater peak beta power. During the neonatal period, cortical excitatory and inhibitory circuits are still maturing, and GABAergic signaling may not yet exert the same stabilizing influence observed in adulthood. Consequently, neural activity may be more strongly shaped by excitatory processes, reflecting a developmental state characterized by heightened plasticity and cortical hyperexcitability (Stedehouder and Kushner, 2016; Sydnor et al., 2025; Wu et al., 2024). Within this framework, the observed associations could indicate that beta oscillatory activity in early life is more closely linked to excitatory neurotransmission than to mature excitation–inhibition balance mechanisms. Conversely, both GABA and Glx levels negatively correlate with relative power in the beta range. These correlations would indicate that higher GABA/Glutamate levels are associated with a greater proportion of beta power. This decrease in relative power could reflect an increase in total broadband power, which would make the proportional contribution of beta smaller (Gonzalez-Carpinteiro et al., 2025).

In contrast to electrophysiological measures, we observed a significant negative association between occipital GABA+ levels and fALFF, indicating that higher GABA+ is associated with reduced infra-slow hemodynamic fluctuations. This relationship mirrors adult findings (Kurcyus et al., 2018; Muthukumaraswamy et al., 2009), and so some aspects of the interaction between GABA-related neurochemical measures and hemodynamic fluctuations may already be present at term age in our sample. These findings raise the possibility that mechanisms linking inhibitory neurochemical processes and cerebral hemodynamics begin to emerge early in development. Indeed, inhibitory interneurons are involved in vascular regulation, including via nitric oxide signaling (Attwell and Iadecola, 2002), which may influence hemodynamic fluctuations before fast electrophysiological and hemodynamic measures become tightly linked (due to the potentially immature role of synaptic GABA in controlling neuronal activity).

Based on adult EEG–fMRI literature, we expected high-frequency EEG activity, particularly gamma-band power or amplitude, to show positive associations with regional ALFF/fALFF, reflecting greater coupling between local electrophysiological activity and spontaneous hemodynamic fluctuations (Palva and Palva, 2012; Scheeringa et al., 2011). In contrast, we identified a significant negative association between gamma center frequency and infra-slow hemodynamic fluctuations in our neonatal sample. Although gamma oscillations are thought to contribute to neurovascular coupling in the mature adult brain (Scheeringa et al., 2011; Sumiyoshi et al., 2012), our findings do not imply that neonatal hemodynamic fluctuations are uncoupled from neural processes altogether (Hendrikx et al., 2019). Rather, they suggest that, in early development, infra-slow hemodynamic fluctuations may not yet show adult-like positive coupling with the specific fast oscillatory features measured here.

A rostral-caudal gradient was observed across analyses. Associations involving GABA+, oscillatory activity, and hemodynamic measures were primarily evident in occipital cortex, whereas frontal regions showed no GABA associations. Instead, the frontal cortex was characterized by associations primarily between oscillatory activity, hemodynamic fluctuations, and Glx levels. This hierarchical development of inhibitory regulation aligns with established models of brain development in which sensory regions mature earlier than association cortices (Basu et al., 2023; Gogtay et al., 2004). Supporting this interpretation, we observed age-related changes in occipital beta center frequency and mALFF but not in frontal cortex, alongside the absence of robust gamma-band features anteriorly. Additionally, the associations found between GABA+ levels and fALFF or beta relative power in the occipital cortex are absent in the frontal lobe. Together, these findings suggest that earlier-maturing sensory systems may exhibit greater correspondence between neurochemical, electrophysiological, and hemodynamic measures than later-maturing frontal association regions. This pattern is consistent with hierarchical models of cortical development, in which primary sensory cortices undergo earlier maturation and functional specialization than higher-order association regions, which continue to develop well beyond birth and support increasingly complex cognitive and behavioral functions (Chomiak and Hu, 2017; Kostović et al., 2019; Sydnor et al., 2025).

An important consideration is that MRS-derived measures (GABA+, Glx) provide indirect estimates of excitatory and inhibitory activity and cannot distinguish between synaptic and neuronal metabolic processes. Furthermore, we refer to GABA+ and Glx concentrations as ‘levels’ (as opposed to absolute concentrations in millimoles), to reflect known and unknown sources of variability. These include inter-scanner differences, relaxation effects, B₀ inhomogeneity, macromolecular contamination, and variation in tissue water content, all of which can influence signal amplitudes and are not fully accounted for, particularly in neonates, where key correction factors (e.g., metabolite relaxation times) and test–retest reliability remain poorly characterized. Nonetheless, our quantification pipeline followed standard best-practice recommendations (Peek et al., 2023) and we have provided clear detail throughout the manuscript on the quantification methods used to facilitate future comparisons.

Regarding the FOOOF analysis, parameters were selected *a priori* based on previously reported settings (Luotonen et al., 2025; Rico-Picó et al., 2023) and were evaluated by maximizing model fit across participants. All included spectra reached a minimum model fit of *R*^2^ = 0.85, irrespective of the presence of clear periodic oscillations. Although this approach supported robust spectral parameterization in the present dataset, we acknowledge that aperiodic exponent estimates may vary with FOOOF parameter settings. Future work in larger neonatal cohorts should systematically assess the sensitivity of aperiodic exponent–neurochemical associations to spectral model configuration.

While prior work has demonstrated associations between MRS-measured GABA and beta center frequency in adults under resting conditions (Baumgarten et al., 2016), indicating that such relationships can be detected in the absence of explicit task demands, many studies examining neurochemical–oscillatory–hemodynamic coupling have used stimulus-evoked paradigms. Due to the methodological challenges of conducting such studies in sleeping infants, our data were acquired at rest, which may differ in sensitivity compared with transient, task-dependent coupling. Notably, excitation-inhibition balance is dynamically modulated by cognitive and sensory demands, with task engagement known to alter MRS derived-GABA and Glx levels, oscillatory activity and neurovascular responses (Bhatia et al., 2019; Fox and Raichle, 2007; Fries, 2015; Pasanta et al., 2022). While implementing multimodal task-based paradigms in infants is challenging, future studies may benefit from combining spontaneous and evoked paradigms to test whether system-level interactions are differentially engaged under task conditions.

Although this study aimed to evaluate general cross-modal coupling in the neonatal brain during rest, vigilance state may influence the observed associations. Neonatal EEG spectral structure, BOLD dynamics, and potentially EEG–fMRI coupling may differ between active sleep, quiet sleep, and wakefulness (Lee et al., 2020; Yang et al., 2026). In the present sample, sleep–wake state was unevenly distributed, with most infants in active sleep, and the sample size was insufficient to robustly assess vigilance-state effects statistically or to perform state-stratified analyses. Therefore, our findings should be interpreted as primarily reflecting EEG–fMRI coupling during a predominantly active-sleep resting condition. Future studies with larger and more balanced samples across vigilance states will be needed to determine whether cross-modal coupling differs between active sleep, quiet sleep, and wakefulness.

Exploration of whether these novel findings extend to preterm neonates is also an important consideration for future research. While unimodal neuroimaging studies have commonly been used to characterize neurobiological development in preterm infants using EEG, MRS or fMRI individually, multimodal studies combining these measures remain scarce, in part due to the considerable practical challenges of neonatal neuroimaging. However, these kinds of studies would be highly valuable as preterm and term infants are likely to be at different stages of neurobiological development. For example, the developmental transition in GABAergic signaling is thought to occur between the third trimester and term age (Tyzio et al., 2008). Furthermore, preterm infants have been reported to exhibit lower GABA and glutamate concentrations than term infants, as well as distinct associations between neurochemical measures and resting-state functional connectivity, which may reflect differences in maturational stage (Kwon et al., 2014). Examining multimodal neurochemical, electrophysiological, and hemodynamic relationships across preterm and term populations may therefore provide further insight into the developmental emergence of these associations and help identify sensitive markers of typical and atypical neurodevelopmental trajectories.

## 5 Conclusion

While adult studies suggest a coherent multiscale E/I system linking EEG, MRS and fMRI measures, our preliminary data demonstrate that this framework may not fully generalize to the neonatal brain, likely due to ongoing and asynchronous spatiotemporal cortical development. This work contributes to improving our understanding of how canonical markers of brain function evolve across development and highlights the importance of multimodal approaches for characterizing early brain development. Further work modeling the impact of dynamically evolving cortical neurobiology on each modality will be required to comprehensively characterize the interdependence of the respective metrics across the neonatal period.

## Data Availability

The raw data supporting the conclusions of this article will be made available by the authors, without undue reservation.
