# Supplementary material for: Neurochemical-hemodynamic-electrophysiological coupling in the neonatal brain: a multimodal MRS-fMRI-EEG investigation
Source: Front Neurosci. 2026 Jul 2;20:1859287. doi: 10.3389/fnins.2026.1859287 (PMC13372886; doi:10.3389/fnins.2026.1859287)
Supplement: Supplementary file 1 [file Data_Sheet_1.pdf]

Supplementary Material

1 Supplementary Figures

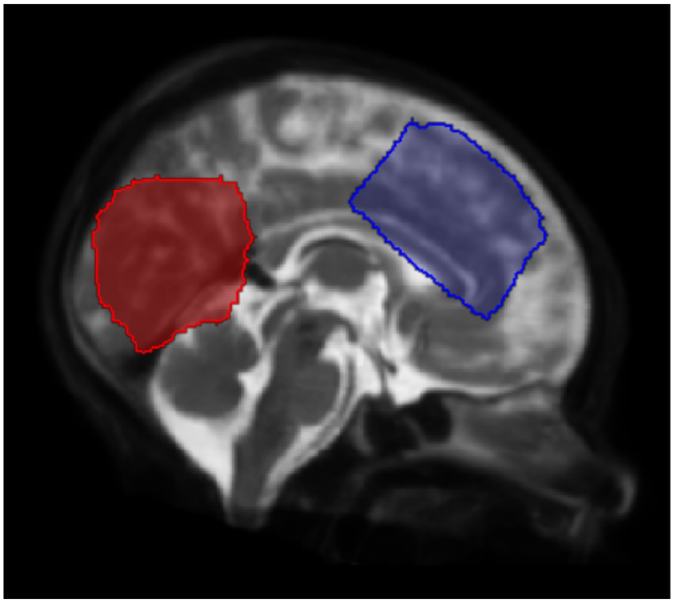

Supplementary Figure 1. Group-level MRS voxel. Group-specific template T2-weighted image overlayed with the group-level MRS voxels in the occipital (red) and frontal (blue) locations.

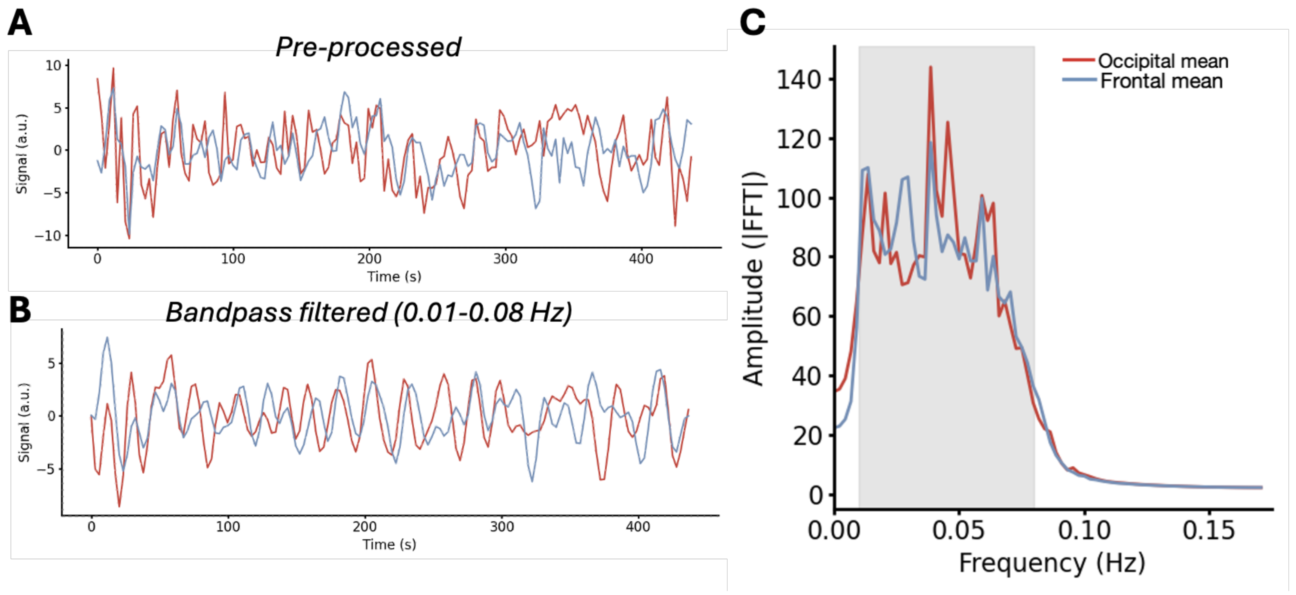

Supplementary Figure 2. fMRI analysis. (A) Exemplar voxel wise mean pre-processed BOLD timeseries extracted from the frontal (blue) and occipital (red) ROIs in a single subject. (B) Bandpassed (0.01-0.08 Hz) BOLD timeseries to extract ALFF in frontal and occipital areas. (C) Power spectral density of the BOLD infra-slow fluctuations. Grey block indicates frequency range of interest (0.01-0.08 Hz) to extract mean and fractional ALFF.

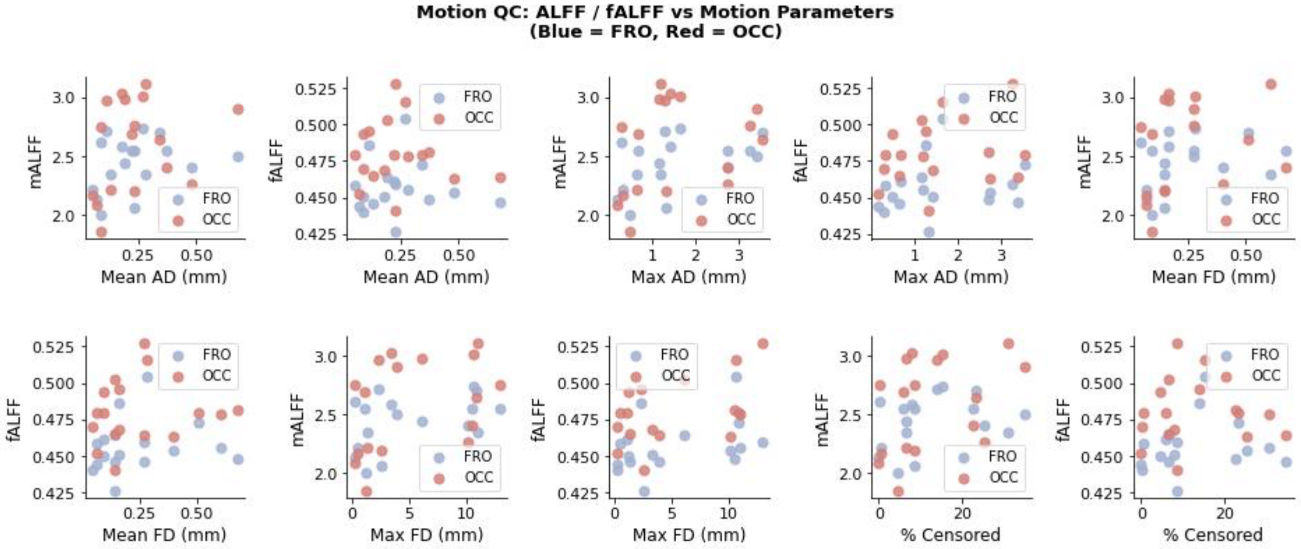

Supplementary Figure 3. No relationship between head motion and infra-slow haemodynamic fluctuations following fMRI data preprocessing. Scatter plots of mean amplitude of low frequency fluctuations (mALFF; top) and fractional ALFF (fALFF; bottom) in the occipital (red) and frontal (blue) regions of interest with head motion parameters (mean and maximum framewise displacement (FD) and absolute displacement (AD), and percentage of censored volumes).

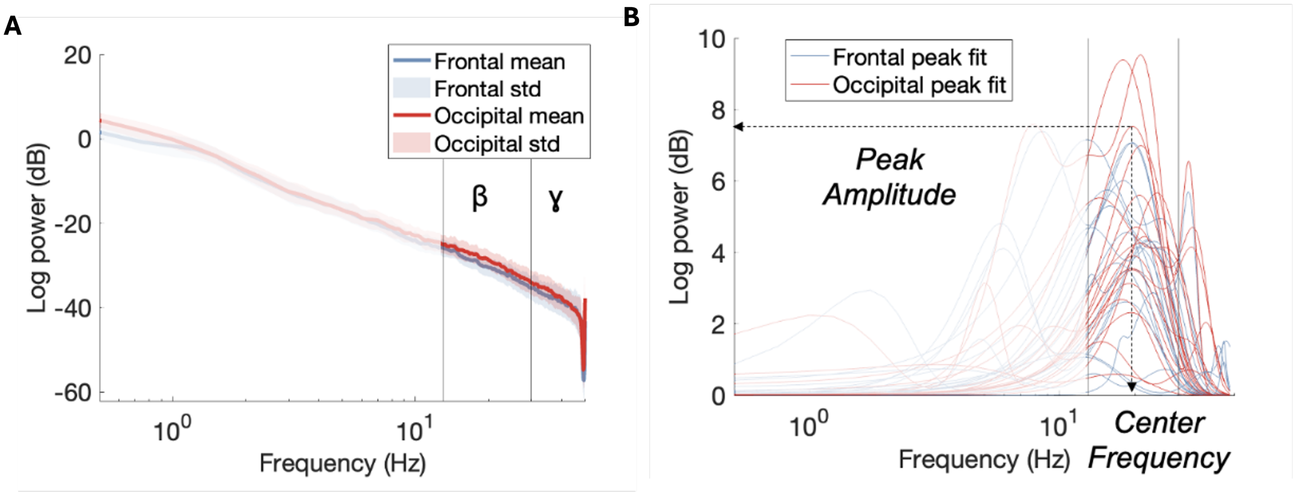

Supplementary Figure 4. **(A)** Power spectral densities (PSD). Mean and standard deviation of PSDs across subjects estimated from the virtual timeseries in the occipital and frontal ROIs. Beta (β) and gamma (γ) frequency ranges of interest highlighted. (**B**) EEG periodic activity parametrisation**.** Periodic features extracted from the SpecParam model fit on each individual subject in each ROI: peak amplitude and centre frequency. Beta and gamma frequency ranges of interest highlighted.

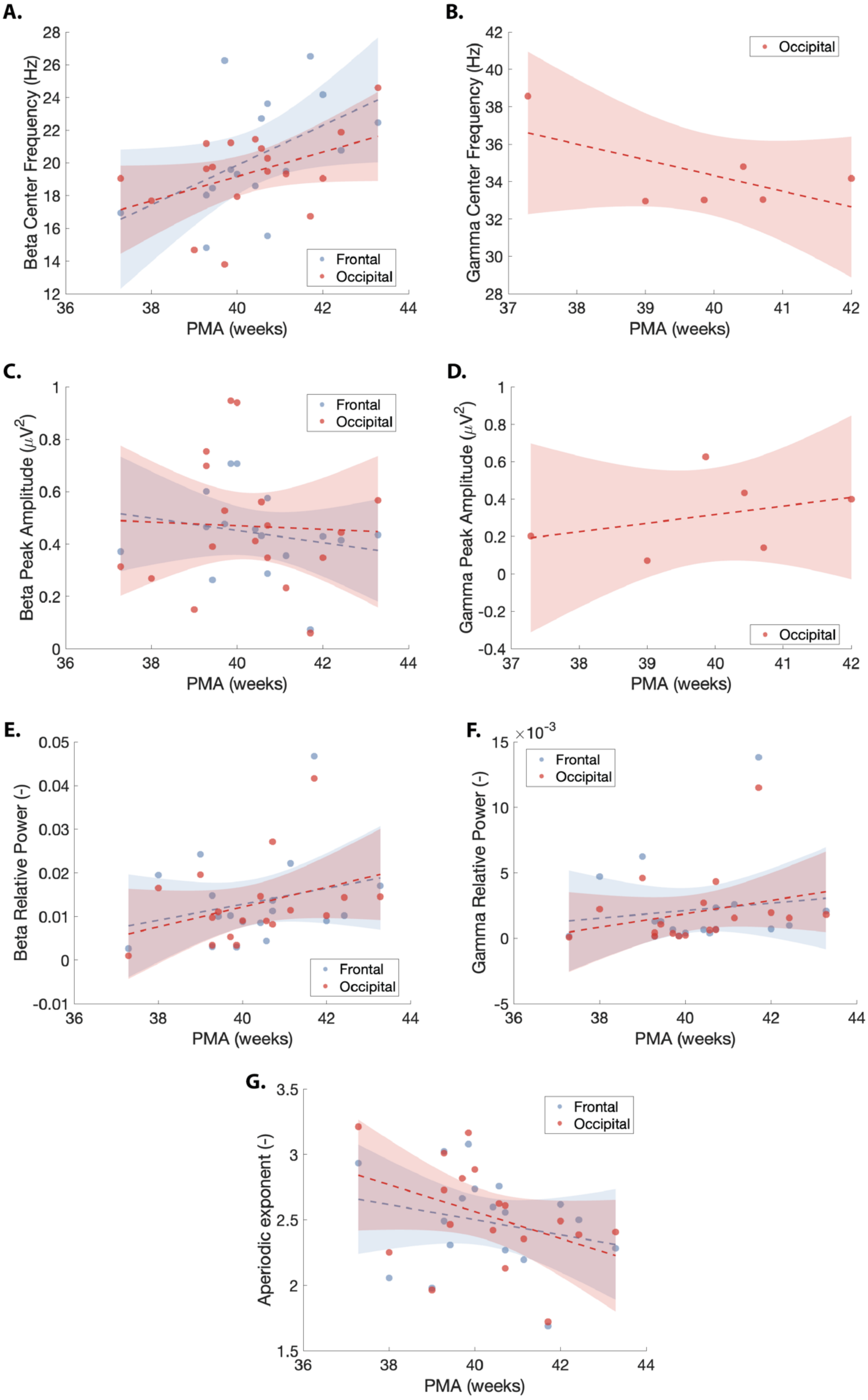
A.
Beta Center Frequency (Hz)
28
26
24
22
20
18
16
14
12
36
38
40
42
44
PMA (weeks)
Frontal
Occipital
B.
Gamma Center Frequency (Hz)
42
40
38
36
34
32
30
28
37
38
39
40
41
42
PMA (weeks)
Occipital
C.
Beta Peak Amplitude (μV^2)
1
0.8
0.6
0.4
0.2
0
36
38
40
42
44
PMA (weeks)
Frontal
Occipital
D.
Gamma Peak Amplitude (μV^2)
1
0.8
0.6
0.4
0.2
0
-0.2
-0.4
37
38
39
40
41
42
PMA (weeks)
Occipital
E.
Beta Relative Power (-)
0.05
0.04
0.03
0.02
0.01
0
-0.01
36
38
40
42
44
PMA (weeks)
Frontal
Occipital
F.
Gamma Relative Power (-)
$\times 10^{-3}$
15
10
5
0
-5
36
38
40
42
44
PMA (weeks)
Frontal
Occipital
G.
Aperiodic exponent (-)
3.5
3
2.5
2
1.5
36
38
40
42
44
PMA (weeks)
Frontal
Occipital

Supplementary Figure 5. PMA effects in EEG periodic and aperiodic components. Scatter plots with overlayed regression lines correlating PMA with beta and gamma center frequency **(A-B),** peak amplitude **(C-D)**, relative power **(E-F)**, and aperiodic exponent **(G). (A,B)** Frontal ROI: n=16 participants, Occipital ROI: n=18 participants, **(E-F,G)** Frontal ROI: n=18 participants, Occipital ROI: n=18 participants, **(B,D)** Occipital ROI: n=6 participants.

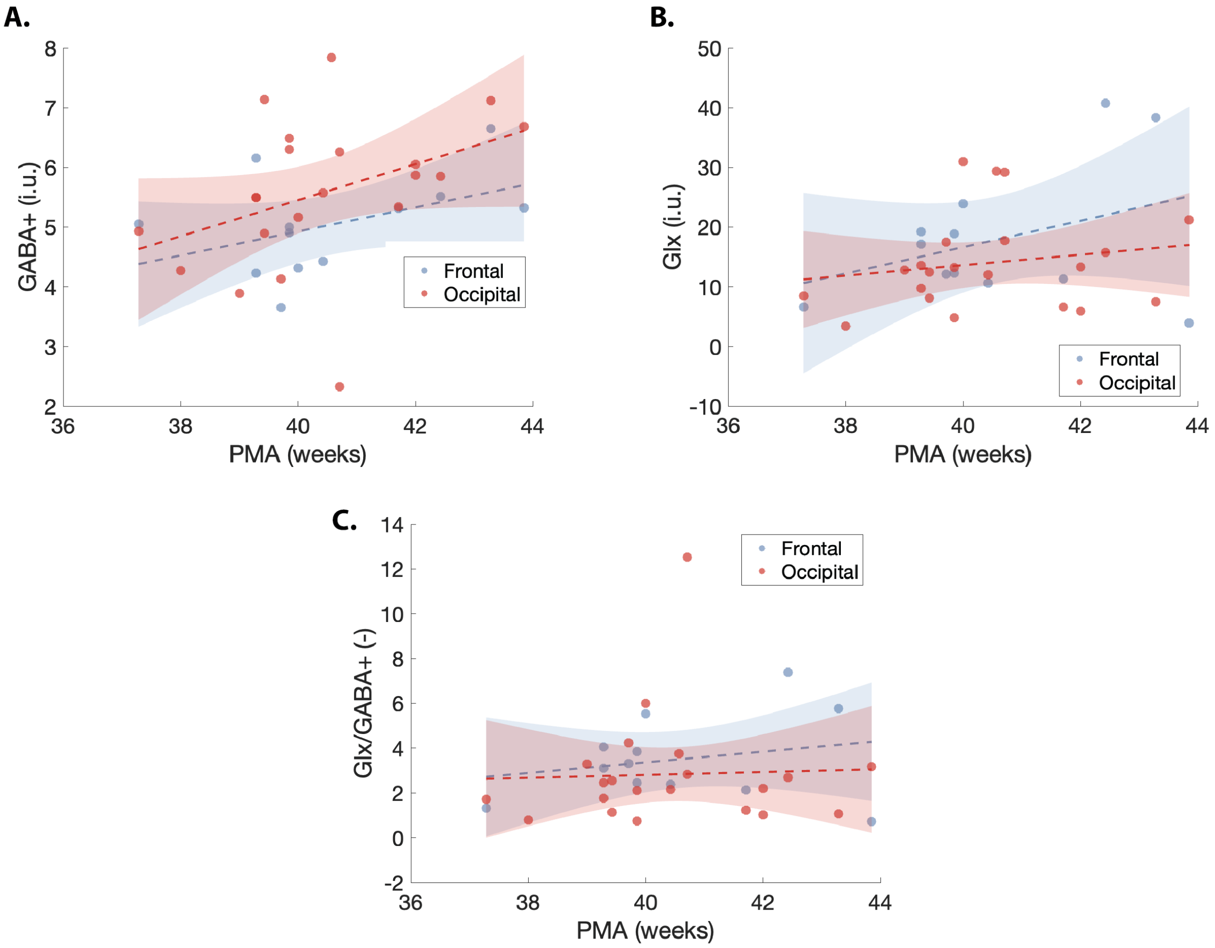

Supplementary Figure 6. PMA effects in MRS data. Scatter plots with overlayed regression lines correlating PMA with GABA+ **(A)**, Glx **(B)** and Glx/GABA+ **(C)**. Frontal ROI: n=12 participants, and occipital ROI: n=21 participants.

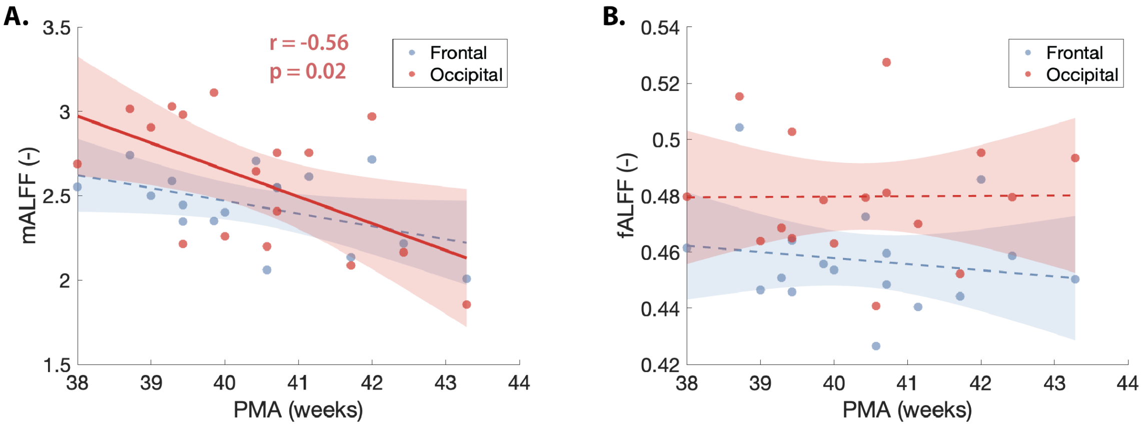

Supplementary Figure 7. PMA effects in fMRI data. Scatter plots with overlayed regression lines correlating mean (**A)** and fractional ALFF (**B)** with PMA. Solid line: $p<0.05$. Mean ALFF significantly negatively correlates with PMA. Frontal and occipital ROIs: n=17 participants.

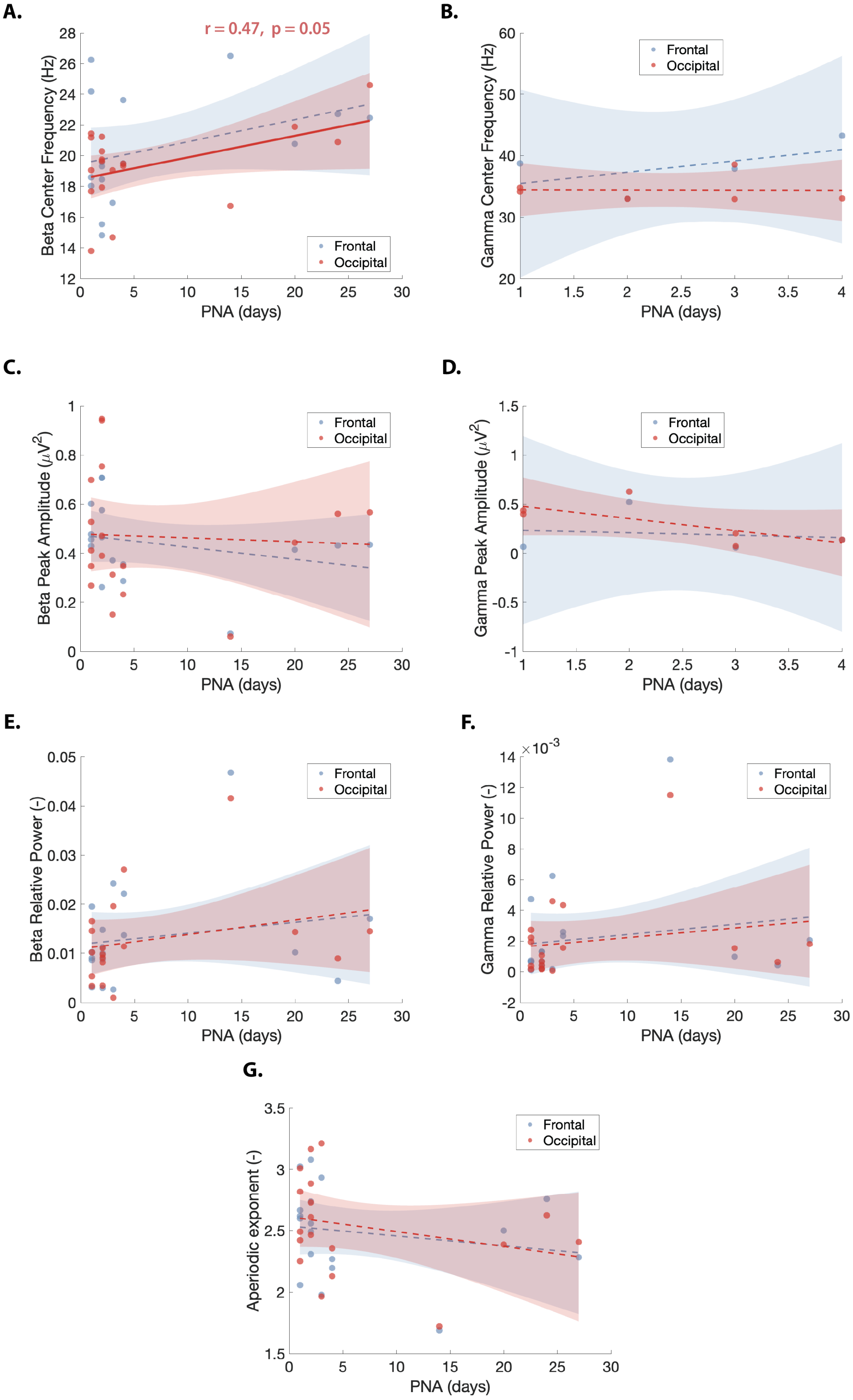
A.
r = 0.47, p = 0.05
Beta Center Frequency (Hz)
PNA (days)
Frontal
Occipital
B.
Gamma Center Frequency (Hz)
PNA (days)
Frontal
Occipital
C.
Beta Peak Amplitude (μV^2)
PNA (days)
Frontal
Occipital
D.
Gamma Peak Amplitude (μV^2)
PNA (days)
Frontal
Occipital
E.
Beta Relative Power (-)
PNA (days)
Frontal
Occipital
F.
$\times 10^{-3}$
Gamma Relative Power (-)
PNA (days)
Frontal
Occipital
G.
Aperiodic exponent (-)
PNA (days)
Frontal
Occipital

Supplementary Figure 8. PNA effects in EEG periodic and aperiodic components. Scatter plots with overlayed regression lines correlating PNA with beta and gamma center frequency (A-B), peak amplitude (C-D), relative power (E-F), and aperiodic exponent (G). (A,B) Frontal ROI: n=16 participants, Occipital ROI: n=18 participants, (E-F,G) Frontal ROI: n=18 participants, Occipital ROI: n=18 participants, (B,D) Occipital ROI: n=6 participants.

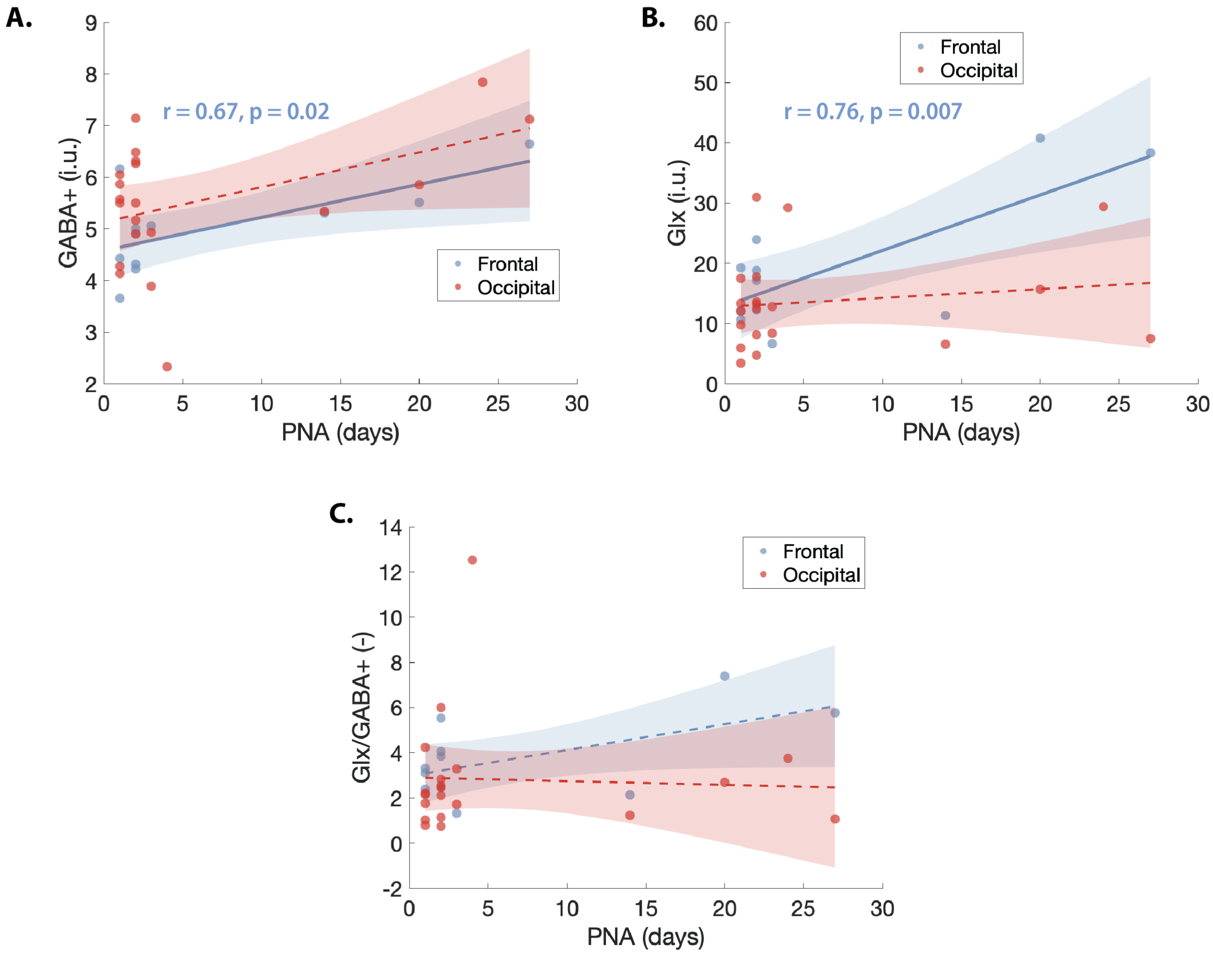

Supplementary Figure 9. PNA effects in MRS data. Scatter plots with overlayed regression lines correlating PNA with GABA+ (**A**), Glx (**B**) and Glx/GABA+ (**C**). Frontal ROI: n=12 participants, and occipital ROI: n=21 participants.

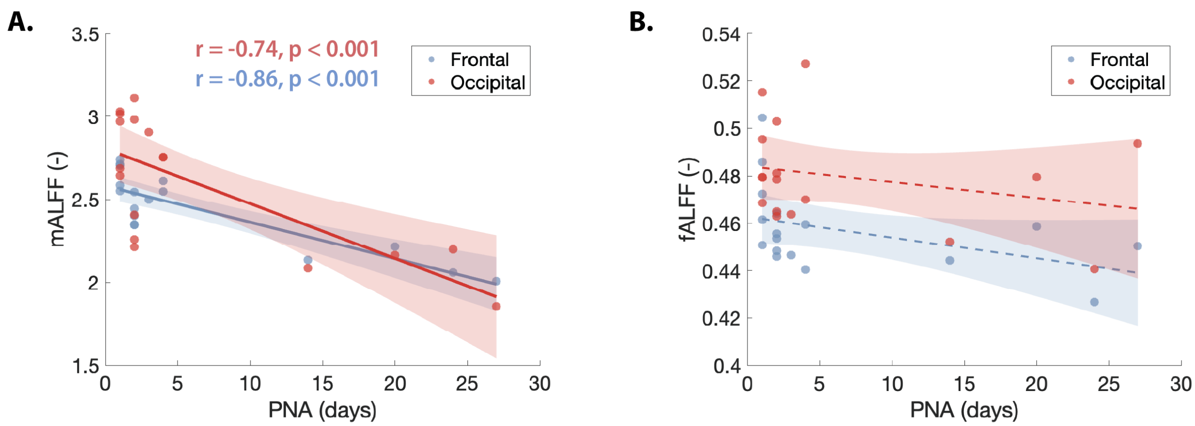

Supplementary Figure 10. PNA effects in fMRI data. Scatter plots with overlayed regression lines correlating mean (**A)** and fractional ALFF (**B)** with PNA. Solid line: p<0.05. Mean ALFF significantly negatively correlates with PMA. Frontal and occipital ROIs: n=17 participants.

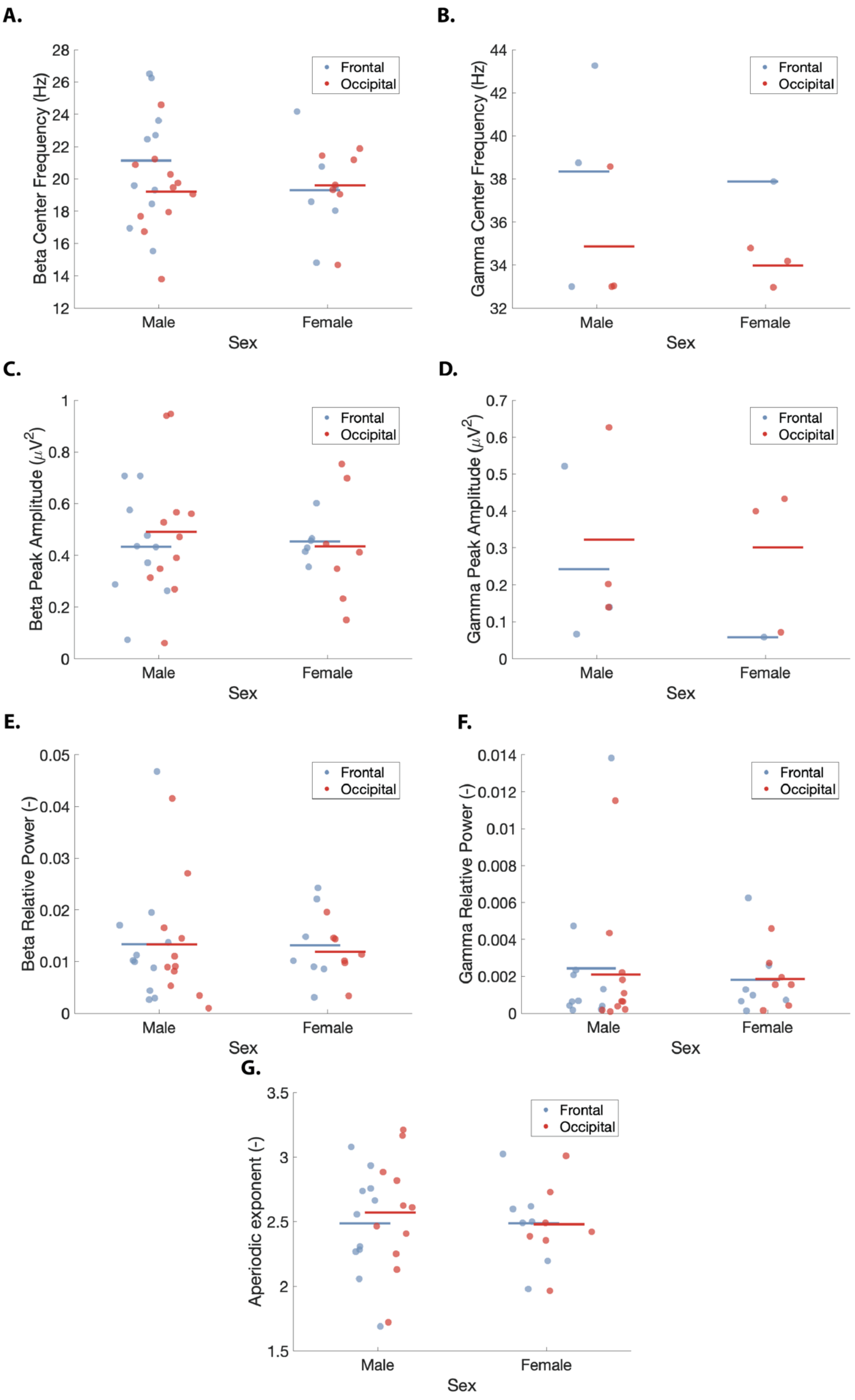
A.
Beta Center Frequency (Hz)
Frontal
Occipital
Male
Female
Sex
B.
Gamma Center Frequency (Hz)
Frontal
Occipital
Male
Female
Sex
C.
Beta Peak Amplitude (μV^2)
Frontal
Occipital
Male
Female
Sex
D.
Gamma Peak Amplitude (μV^2)
Frontal
Occipital
Male
Female
Sex
E.
Beta Relative Power (-)
Frontal
Occipital
Male
Female
Sex
F.
Gamma Relative Power (-)
Frontal
Occipital
Male
Female
Sex
G.
Aperiodic exponent (-)
Frontal
Occipital
Male
Female
Sex

Supplementary Figure 11. Sex effects in EEG periodic and aperiodic components. Scatter plots with overlayed regression lines correlating sex with beta and gamma center frequency (A-B), peak amplitude (C-D), relative power (E-F), and aperiodic exponent (G). (A,B) Frontal ROI: n=16 participants, Occipital ROI: n=18 participants, (E-F,G) Frontal ROI: n=18 participants, Occipital ROI: n=18 participants, (B,D) Occipital ROI: n=6 participants.

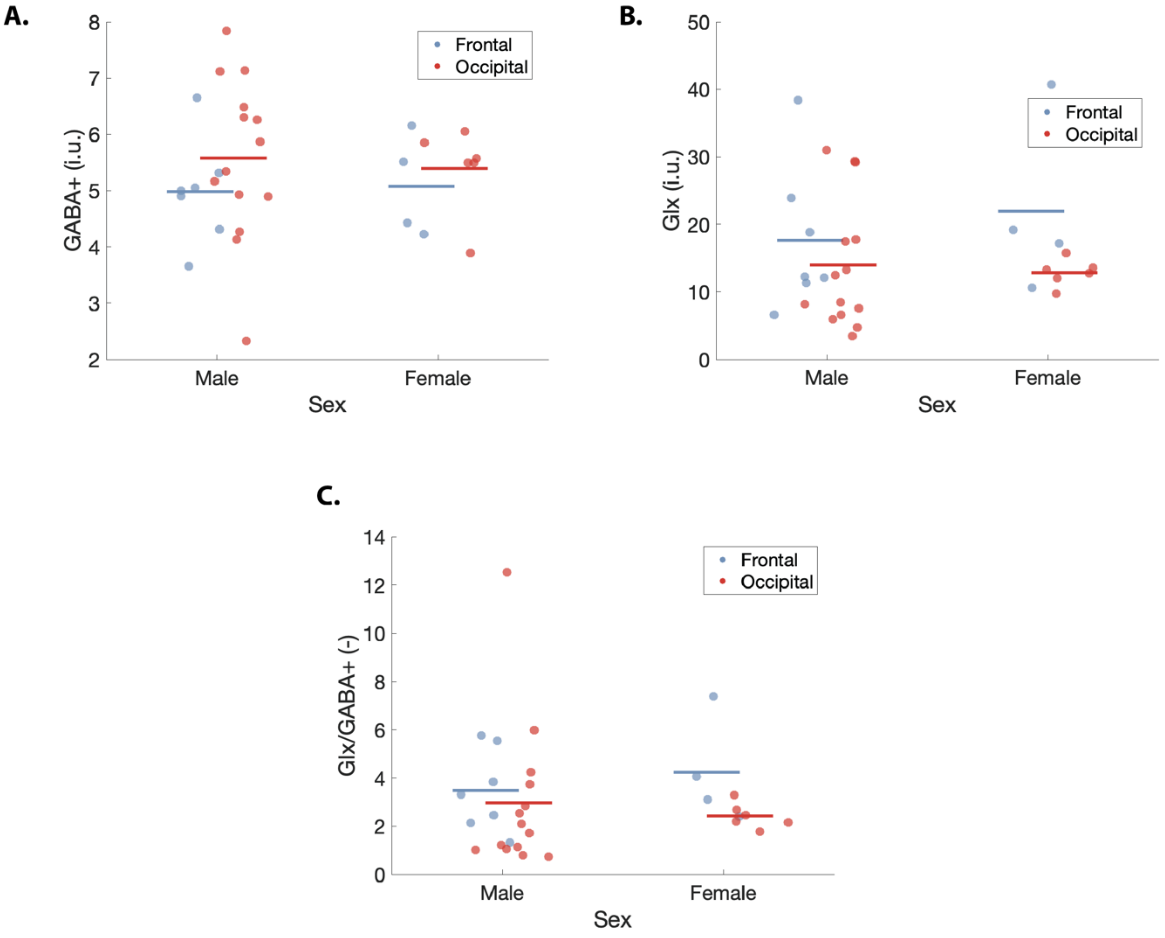

Supplementary Figure 12. Sex effects in MRS data. Scatter plots with overlayed regression lines correlating sex with GABA+ (**A**), Glx (**B**) and Glx/GABA+ (**C**). Frontal ROI: n=12 participants, and occipital ROI: n=21 participants.

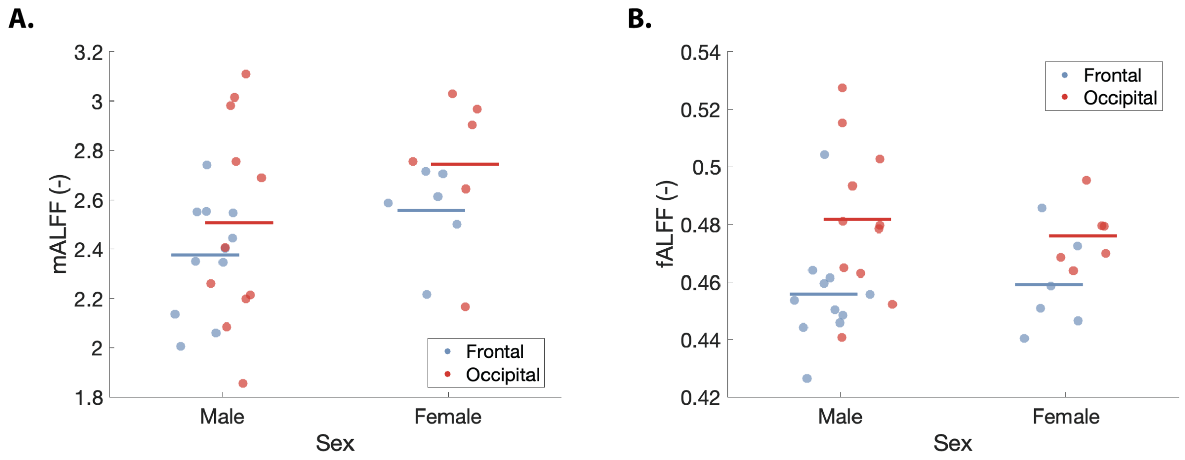

Supplementary Figure 13. Sex effects in fMRI data. Scatter plots with overlayed regression lines correlating mean (**A)** and fractional ALFF (**B)** with sex. Solid line: $p<0.05$. Mean ALFF significantly negatively correlates with PMA. Frontal and occipital ROIs: n=17 participants.

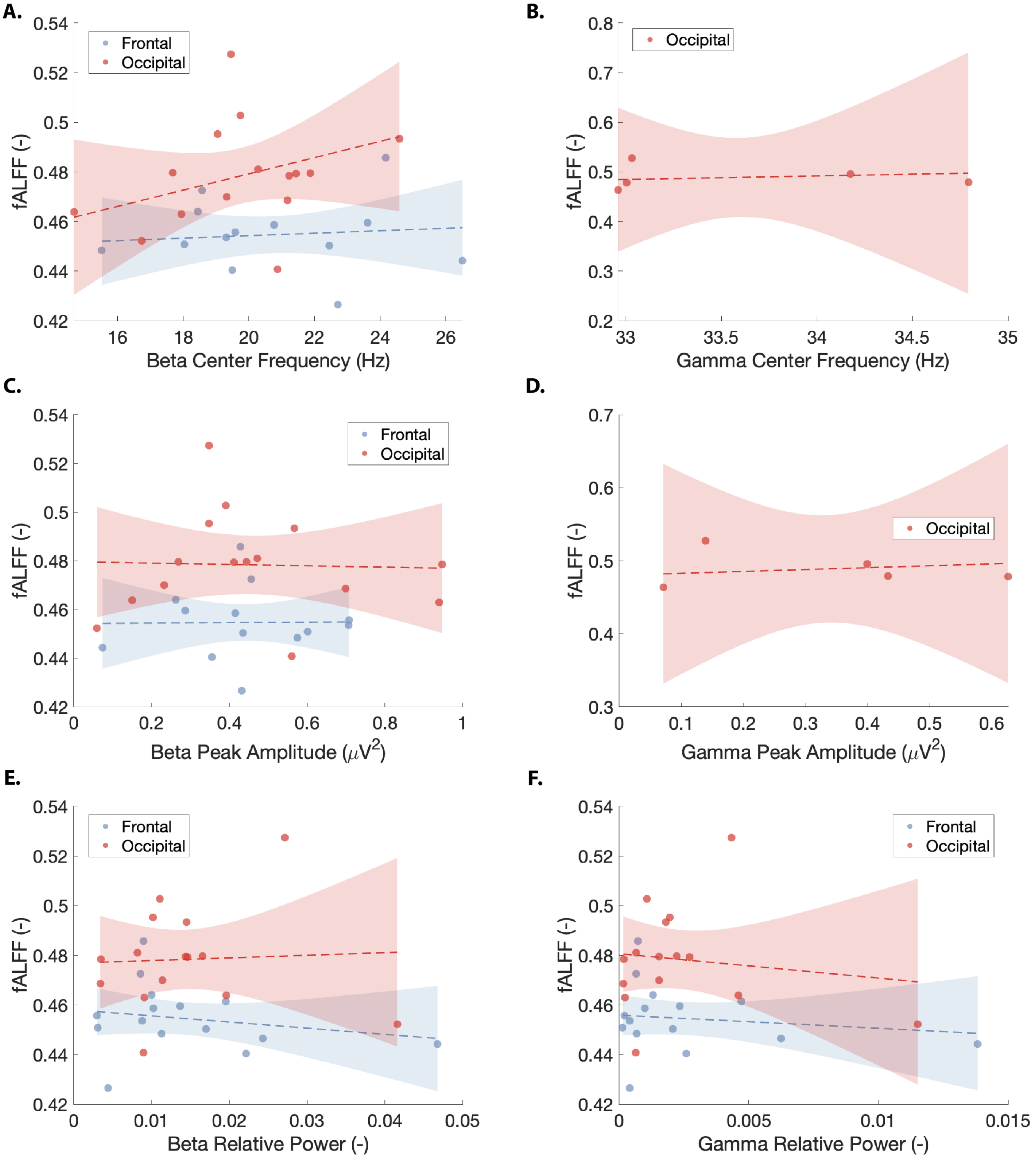

Supplementary Figure 14. fALFF does not significantly correlate with beta or gamma relative power, centre frequency or peak amplitude. Scatter plots with overlayed regression lines correlating fALFF to EEG beta and gamma center frequency **(A-B),** peak amplitude **(C-D)**, relative power **(E-F;** n=15 participants). (**A, C**) Frontal ROI: n=13 participants, occipital ROI: n=15 participants, and (**B, D**) occipital ROI: n=5 participants.

2 Supplementary Tables

Metabolite	T1_GM	T1_WM	T2_GM	T2_WM
**ASC**	1340	1190	(125+105)/2	172
**ASP**	1340	1190	(111+190)/2	148
**CR**	1460	1240	(148+144)/2	166
**GABA**	1310	1310	(102+75)/2	(102+75)/2
**GLC**	1340	1190	(117+88)/2	155
**GLN**	1340	1190	(122+99)/2	168
**GLU**	1270	1170	(135+122)/2	124
**GLY**	1340	1190	(102+81)/2	152
**GPC**	1300	1080	(274+222)/2	218
**GSH**	1340	1190	(100+77)/2	145
**LAC**	1340	1190	(110+99)/2	159
**mI**	1230	1010	(244+229)/2	161
**NAA**	1470	1350	(253+263)/2	343
**NAAG**	1340	1190	(128+107)/2	185
**PCH**	1300	1080	(274+221)/2	213
**PCR**	1460	1240	(148+144)/2	166
**PE**	1340	1190	(119+86)/2	158
**SCY**	1340	1190	(125+107)/2	170
**TAU**	1340	1190	(123+102)/2	(123+102)/2
**tNAA**	(1470+1340)/2	(1350+1190)/2	(253+263+128+107)/4	(343+185)/2
**tCr**	(1460+1460)/2	(1240+1240)/2	(148+144+148+144)/4	(166+166)/2
**tCho**	(1300+1080)/2	(1080+1080)/2	(274+222+274+221)/4	(218+213)/2
**Glx**	(1340+1270)/2	(1190+1170)/2	(122+99+135+122)/4	(168+124)/2
**water**	1331	832	110	79.2

Supplementary Table 1. Tissue-specific metabolite and water T1 and T2 relaxation times used for tissue-correction of water-scaled metabolite concentrations according to (Gasparovic et al., 2006). T1 for GABA was obtained from (Puts et al., 2013). Water relaxation values were obtained from (Lu et al., 2005).

	SNR	FWHM	Residual water	Frequency shift	Residual fit	fGM	fWM	fCSF
OCC	170.74 (61.61)	6.61 (1.00)	0.96 (0.19)	-2.40 (0.79)	6.88 (7.44)	0.42 (0.18)	0.42 (0.11)	0.11 (0.08)
Frontal	154.10 (52.12)	6.62 (5.09)	1.02 (0.28)	-2.26 (.11)	10.84 (9.61)	0.35 (0.10)	0.45 (0.17)	0.10 (0.07)

Supplementary Table 2. MRS data quality metrics. Medians (interquartile range (IQR)) are shown. Quality metrics include signal to noise ratio (SNR; creatine peak), full-width half maximum (FWHM; Hertz (Hz); creatine peak), fit residuals (edit-OFF and difference spectra) and frequency shift (Hz). Gray matter (GM), white matter (WM) and cerebral spinal fluid (CSF) voxel fractions are also shown. Frontal voxel: n=12 participants and occipital voxel: n=21 participants.

Modality	Measure	N
MRS	GABA+	Frontal: 12<br>Occipital: 21
	Glx	Frontal: 12<br>Occipital: 21
	Glx/GABA+	Frontal: 12<br>Occipital: 21
EEG	Beta relative power	Frontal: 18<br>Occipital: 18
	Beta centre frequency	Frontal: 16<br>Occipital: 18
	Beta peak amplitude	Frontal: 16<br>Occipital: 18
	Gamma relative power	Frontal: 18<br>Occipital:18
	Gamma centre frequency	Frontal: 4<br>Occipital: 6
	Gamma peak amplitude	Frontal: 4<br>Occipital: 6
	Aperiodic exponent	Frontal: 18<br>Occipital: 18

fMRI	Mean ALFF	Frontal: 17<br>Occipital: 17
	Fractional ALFF	Frontal: 17<br>Occipital: 17

Supplementary Table 3. MRS, EEG and fMRI measures of interest and associated sample sizes. Frontal and occipital ROIs do not necessarily have the same sample size due to separate MRS acquisitions, data quality and depending on the FOOOF model fit.

Measure	Covariate	ROI	Effect	Pval	N
Beta Relative Power	PMA	Frontal	0.26	0.290	18
		Occipital	0.36	0.141	18
	PNA	Frontal	0.18	0.471	18
		Occipital	0.26	0.290	18
	Sex	Frontal	-0.00	0.966	18
		Occipital	-0.00	0.762	18
Beta Centre Frequency	PMA	Frontal	0.50	0.050	16
		Occipital	0.44	0.069	18
	PNA	Frontal	0.36	0.165	16
		Occipital	0.47	0.050*	18
	Sex	Frontal	-1.83	0.336	16
		Occipital	0.38	0.773	18
Beta Peak Amplitude	PMA	Frontal	-0.21	0.436	16
		Occipital	-0.04	0.866	18
	PNA	Frontal	-0.27	0.305	16
		Occipital	-0.05	0.830	18
	Sex	Frontal	0.02	0.812	16
		Occipital	-0.06	0.651	18
Gamma Relative Power	PMA	Frontal	0.13	0.606	18

		Occipital	0.28	0.252	18
	PNA	Frontal	0.17	0.499	18
		Occipital	0.20	0.437	18
	Sex	Frontal	-0.00	0.708	18
		Occipital	-0.00	0.855	18
Gamma Centre Frequency	PMA	Frontal	0.25	0.752	4
		Occipital	-0.63	0.184	6
	PNA	Frontal	0.57	0.434	4
		Occipital	-0.02	0.975	6
	Sex	Frontal	-0.46	0.945	4
		Occipital	-0.89	0.667	6
Gamma Peak Amplitude	PMA	Frontal	0.40	0.595	4
		Occipital	0.35	0.494	6
	PNA	Frontal	-0.14	0.855	4
		Occipital	-0.71	0.114	6
	Sex	Frontal	-0.18	0.581	4
		Occipital	-0.02	0.916	6
Aperiodic exponent	PMA	Frontal	-0.24	0.346	18
		Occipital	-0.39	0.106	18
	PNA	Frontal	-0.19	0.461	18

		Occipital	-0.26	0.294	18
	Sex	Frontal	0.00	0.995	18
		Occipital	-0.09	0.646	18

Supplementary Table 4. Demographic effects in EEG periodic and aperiodic components. Linear associations between demographic variables, PMA, PNA, and Sex, and each EEG measure. For PMA and PNA, Effect represents the correlation coefficient. For Sex, coded as 0 = male and 1 = female, Effect represents the linear model coefficient, corresponding to the estimated female–male difference. *$p < 0.05$.

Measure	Covariate	ROI	Effect	Pval	N
GABA+	PMA	Frontal	0.47	0.140	11
		Occipital	0.35	0.133	20
	PNA	Frontal	0.67	0.023*	11
		Occipital	0.44	0.052	20
	Sex	Frontal	0.10	0.871	11
		Occipital	-0.19	0.772	20
Glx	PMA	Frontal	0.74	0.010*	11
		Occipital	0.10	0.690	20
	PNA	Frontal	0.76	0.007*	11
		Occipital	0.15	0.525	20
	Sex	Frontal	4.29	0.569	11
		Occipital	-1.09	0.789	20
Glx/GABA+	PMA	Frontal	0.64	0.035*	11
		Occipital	0.03	0.899	20
	PNA	Frontal	0.57	0.065	11
		Occipital	-0.05	0.831	20
	Sex	Frontal	0.76	0.535	11
		Occipital	-0.55	0.681	20

Supplementary Table 5. Demographic effects in MRS measures. Linear associations between demographic variables, PMA, PNA, and Sex, and each MRS measure. For PMA and PNA, Effect

represents the correlation coefficient. For Sex, coded as 0 = male and 1 = female, Effect represents the linear model coefficient, corresponding to the estimated female–male difference. *p < 0.05.

Measure	Covariate	ROI	Effect	Pval	N
mean ALFF	PMA	Frontal	-0.47	0.056	17
		Occipital	-0.56	0.018*	17
	PNA	Frontal	-0.86	<0.001*	17
		Occipital	-0.74	<0.001*	17
	Sex	Frontal	0.18	0.119	17
		Occipital	0.24	0.250	17
fractional ALFF	PMA	Frontal	-0.17	0.515	17
		Occipital	0.01	0.975	17
	PNA	Frontal	-0.43	0.085	17
		Occipital	-0.27	0.300	17
	Sex	Frontal	0.00	0.731	17
		Occipital	-0.01	0.630	17

Supplementary Table 6. Demographic effects in ALFF features. Linear associations between demographic variables, PMA, PNA, and Sex, and each ALFF measure. For PMA and PNA, Effect represents the correlation coefficient. For Sex, coded as 0 = male and 1 = female, Effect represents the linear model coefficient, corresponding to the estimated female–male difference. *p < 0.05.

Measure 1	Measure 2	ROI	Rho	Pval	N
GABA+	Beta Relative Power	Frontal	-0.23	0.59	10
		Occipital	-0.64	0.01*	17
	Beta Centre Frequency	Frontal	-0.29	0.48	10
		Occipital	0.46	0.09	17
	Beta Peak Amplitude	Frontal	0.28	0.50	10
		Occipital	0.48	0.07	17
	Gamma Relative Power	Frontal	-0.08	0.85	10
		Occipital	-0.49	0.06	17
	Gamma Centre Frequency	Occipital	-0.14	0.86	6
	Gamma Peak Amplitude	Occipital	0.78	0.22	6
Glx	Beta Relative Power	Frontal	-0.78	0.02*	10
		Occipital	-0.14	0.61	17
	Beta Centre Frequency	Frontal	-0.57	0.14	10
		Occipital	-0.11	0.68	17
	Beta Peak Amplitude	Frontal	0.87	0.005*	10
		Occipital	0.39	0.15	17
	Gamma Relative Power	Frontal	-0.79	0.02*	10
		Occipital	-0.28	0.31	17
	Gamma Centre Frequency	Occipital	0.17	0.83	6

	Gamma Peak Amplitude	Occipital	0.40	0.60	6

Supplementary Table 7. Linear partial correlation results between MRS neurotransmitter concentrations and EEG high-frequency components. Partial correlation coefficients are reported after controlling for PMA and PNA. Only analyses with sufficient available data are shown. *p<0.05.

Measure 1	Measure 2	ROI	Rho	Pval	N
Glx/GABA+	Aperiodic exponent	Frontal	0.60	0.12	10
		Occipital	-0.12	0.66	17

Supplementary Table 8. Linear partial correlation results between MRS neurotransmitter ratio and EEG aperiodic exponent. Partial correlation coefficients are reported after controlling for PMA and PNA.

Measure 1	Measure 2	ROI	Rho	Pval	N
GABA+	mean ALFF	Frontal	0.65	0.24	7
		Occipital	-0.18	0.56	15
	fractional ALFF	Frontal	0.33	0.58	7
		Occipital	-0.55	0.05*	15
Glx	mean ALFF	Frontal	0.29	0.64	7
		Occipital	-0.02	0.95	15
	fractional ALFF	Frontal	0.67	0.21	7
		Occipital	0.05	0.87	15

Supplementary Table 9. Linear partial correlation results between MRS neurotransmitter concentrations and fMRI ALFF measures. Partial correlation coefficients are reported after controlling for PMA and PNA. *$p < 0.05$.

Measure 1	Measure 2	ROI	Rho	Pval	N
mean ALFF	Beta Relative Power	Frontal	-0.23	0.45	15
		Occipital	-0.30	0.32	15
	Beta Centre Frequency	Frontal	-0.21	0.54	13
		Occipital	0.25	0.41	15
	Beta Peak Amplitude	Frontal	-0.05	0.90	13
		Occipital	0.01	0.98	15
	Gamma Relative Power	Frontal	-0.30	0.32	15
		Occipital	-0.22	0.47	15
	Gamma Centre Frequency	Occipital	-1.00	0.02*	5
	Gamma Peak Amplitude	Occipital	0.49	0.67	5
fractional ALFF	Beta Relative Power	Frontal	-0.26	0.40	15
		Occipital	0.05	0.86	15
	Beta Centre Frequency	Frontal	0.12	0.72	13
		Occipital	0.35	0.24	15
	Beta Peak Amplitude	Frontal	0.02	0.96	13
		Occipital	-0.04	0.90	15
	Gamma Relative Power	Frontal	-0.18	0.56	15
		Occipital	-0.15	0.63	15
	Gamma Centre Frequency	Occipital	0.43	0.72	5
	Gamma Peak Amplitude	Occipital	0.55	0.63	5

Supplementary Table 10. Linear partial correlation results between fMRI ALFF measures and EEG high-frequency components. Partial correlation coefficients are reported after controlling for PMA and PNA. Only analyses with sufficient available data are shown. *$p < 0.05$.
